# Fluorescence-based biosensing with DNA nanostructures and DNA origami: from programmable architectures to quantitative readouts

**DOI:** 10.1186/s11671-026-05083-7

**Published:** 2026-11-04

**Authors:** Seoyoung Kim, Min Yu Choi, Hyoyoung Jeong, Ha Rim Ahn, Jin-Ha Choi

**Affiliations:** 1 School of Chemical Engineering, Jeonbuk National University, 567 Baekje- daero, Deokjin-gu, Jeonju-si, 54896 Jeonbuk State Republic of Korea; 2 Electrical and Computer Engineering, University of California, Davis, One Shields Avenue, Davis, CA 95616 United States of America; 3 Department of Surgery, Jeonbuk National University Medical School, Jeonju-si, 54907 Republic of Korea

**Keywords:** DNA nanostructure, DNA origami, Fluorescence biosensing, Förster resonance energy transfer (FRET), Plasmon-enhanced fluorescence (PEF)

## Abstract

DNA nanotechnology and DNA origami have emerged as promising platforms for biosensing by exploiting the programmability of DNA sequences and structural self-assembly. Diverse DNA nanostructures, including three-way junctions (TWJs), dendritic assemblies, hydrogels, and tetrahedral DNA nanostructures (TDNs), provide structurally defined frameworks for the organization of functional components. DNA origami further enables highly programmable and precise spatial positioning by folding long DNA scaffolds with multiple staple strands. Their high biocompatibility and nanoscale spatial addressability allow the controlled organization of fluorophores, quenchers, aptamers, nanoparticles, and other functional components, which is particularly advantageous for fluorescence-based sensing strategies that rely on precise spatial control of these components. In this review, we first introduce the fundamental principles of fluorescence-based biosensing, including Förster resonance energy transfer (FRET) and plasmon-enhanced fluorescence (PEF). Then categorize DNA nanostructure-based fluorescence biosensors according to their structural architectures and sensing mechanisms. DNA origami-based platforms are subsequently classified based on their signal transduction strategies. Representative studies are discussed in terms of their design principles and sensing performance. We further critically discuss the remaining challenges, including structural stability, scalability, signal reproducibility, and clinical translation, and provide perspectives on the future development of next-generation fluorescence biosensing platforms enabled by programmable DNA architectures.

## Introduction

The sensitive and specific detection and quantification of biological targets are fundamental to clinical diagnostics, environmental monitoring, and biomedical research [1–3]. Among various analytical approaches, fluorescence-based detection has long served as a cornerstone of biosensing owing to its high sensitivity, broad dynamic range, multiplexing capability, and compatibility with both solution-based assays and bioimaging applications [4, 5]. Despite these advantages, conventional fluorescence biosensors often suffer from limited sensitivity and specificity, as well as performance degradation in complex biological environments. Over the past four decades, DNA nanotechnology has emerged as a promising strategy to address these challenges by providing programmable nanoscale platforms for the rational design of biosensing architectures [6].

DNA nanotechnology exploits the high predictability and specificity of Watson–Crick base pairing to construct structurally well-defined nanostructures with precise control over geometry, dimensionality, and the spatial placement of functional components [7]. Since Nadrian C. Seeman first proposed the concept of using DNA as a structural material in the early 1980s, the field has evolved from simple branched junctions to tile-based assemblies, scaffolded DNA origami, and sophisticated two- and three-dimensional architectures formed through supramolecular polymerization [8]. Among these advances, DNA origami, introduced by Paul Rothemund in 2006 [9], has become one of the most powerful platforms, enabling a long single-stranded DNA scaffold to be folded into virtually arbitrary shapes while allowing hundreds of individual sites to be addressed with sub-nanometer precision [10, 11].

The exceptional spatial programmability of DNA nanostructures and DNA origami enables the precise arrangement of functional elements, including fluorophores, quenchers, aptamers, nanoparticles, and enzymes [12, 13]. Such programmable organization significantly enhances signal transduction efficiency and signal amplification while enabling tunable optical responses toward target analytes, thereby providing versatile platforms for fluorescence biosensing [14]. Furthermore, precise spatial control improves fluorescence signal generation and amplification, facilitating quantitative analysis, multiplexed detection, and ultrasensitive biosensing that are difficult to achieve using conventional fluorescence-based platforms [15, 16]. These unique capabilities have also led to the development of diverse fluorescence signal transduction mechanisms, including Förster resonance energy transfer (FRET) [17, 18], fluorescence quenching and recovery [19], de novo fluorescence generation [20, 21], and plasmon-enhanced fluorescence (PEF) [22, 23]. Collectively, these advances have transformed DNA nanotechnology from a passive structural scaffold into a programmable platform capable of actively regulating fluorescence signals for highly sensitive and selective biosensing.

Recent reviews have highlighted the potential of structurally programmable DNA nanostructures for biosensing applications. Previous studies have reviewed specific DNA nanostructures, including framework DNA [24], TWJs [25], and TDNs [26, 27], as well as broader DNA nanostructure-based sensing interfaces and cellular imaging platforms [28]. Other reviews have addressed DNA nanotechnology-based biosensors from broader perspectives, including molecular recognition, signal amplification, and emerging platforms that integrate DNA origami and Clustered Regularly Interspaced Short Palindromic Repeats (CRISPR) technologies [29]. DNA origami has also been reviewed as a versatile platform for biosensing and nanoscale molecular organization, covering fluorescence, SERS, electrochemical, and microscopy-based sensing [30–35]. However, existing reviews have generally focused on individual DNA nanostructures, broad biological applications, or diverse biosensing modalities. In contrast, the present review specifically focuses on fluorescence biosensing using diverse DNA nanostructures and DNA origami, with particular emphasis on the relationship between nanostructural morphology, spatial organization, and fluorescence signal modulation. By comparing these structural and fluorescence characteristics, this review provides a framework for understanding how DNA nanostructure selection influences sensing performance and for selecting appropriate architectures for fluorescence biosensing applications.

In this review, we first summarize fluorescence biosensors based on representative DNA nanostructures, excluding DNA origami, by classifying them into six categories: three-way junctions (TWJs), hyperbranched and dendritic assemblies, tetrahedral DNA nanostructures (TDNs), DNA hydrogels, other spatially organized scaffolds, and dynamic DNA nanostructures. For each category, we discuss the underlying design principles and fluorescence signal generation strategies. We then review DNA origami-based fluorescence biosensors according to their signal transduction mechanisms, including PEF, FRET-based sensing, and non-FRET fluorescence sensing. Finally, we highlight the current technical challenges and future research directions, providing perspectives on the continued development of DNA nanostructure-based fluorescence biosensing platforms.

## Fluorescence biosensing

Fluorescence biosensors provide highly sensitive and versatile analytical platforms by converting molecular recognition events into measurable changes in fluorescence signals [36]. In general, a fluorescence biosensor consists of two key components: a recognition element that specifically binds the target analyte and a signal transduction module that translates the recognition event into a fluorescence response [5]. Recognition elements include complementary nucleic acid strands, aptamers, DNAzymes, antibodies, enzymes, and other biorecognition molecules, whereas signal transduction relies on photophysical mechanisms such as FRET, nanosurface energy transfer (NSET), photoinduced electron transfer (PET), collisional quenching, and plasmonic enhancement. In this section, we briefly introduce the fundamental principles of the fluorescence signal transduction mechanisms most widely employed in DNA nanostructure- and DNA origami-based biosensing.

FRET is a non-radiative energy transfer process in which excitation energy is transferred from an excited donor fluorophore to a ground-state acceptor chromophore through dipole–dipole coupling. The FRET efficiency depends on both the donor–acceptor distance and the Förster radius, decreasing with the sixth power of the intermolecular distance (r^6^). Consequently, FRET is highly sensitive to distance changes within approximately 2–10 nm, making it an ideal molecular ruler for monitoring nanoscale structural changes [37]. Accordingly, FRET-based biosensors are typically designed by positioning donor and acceptor fluorophores at predetermined locations such that target recognition induces a conformational change, thereby altering the donor–acceptor separation and FRET efficiency [38, 39]. A widely used implementation of FRET is ratiometric fluorescence sensing, in which two or more fluorescence signals are measured simultaneously, and their intensity ratio is used as the analytical output [17, 40]. Because the signal is internally referenced, ratiometric measurements effectively compensate for variations in probe concentration, excitation intensity, and environmental conditions, thereby substantially improving the accuracy and robustness of quantitative analysis, particularly in complex biological samples.

DNA nanostructure- and DNA origami-based fluorescence biosensors employ a variety of fluorescence signal transduction mechanisms, including FRET, NSET, PET, and collisional quenching, to convert target recognition into measurable fluorescence changes. In these systems, fluorescence is suppressed (turn-off) when a fluorophore and a quencher are brought into close proximity, whereas fluorescence is restored (turn-on) when target binding separates the fluorophore from the quencher [41–43]. Turn-on sensing has been widely adopted because of its low background signal and straightforward signal interpretation. Representative examples include fluorophore- and quencher-labeled molecular beacons [43, 44], fluorescently labeled single-stranded DNA adsorbed onto graphene oxide or positioned in close proximity to gold nanoparticles (AuNPs) [43, 45, 46], and small-molecule fluorescent dyes that become emissive upon intercalation into double-stranded DNA [41].

PEF utilizes the localized surface plasmon resonance (LSPR) of metallic nanostructures to substantially amplify the emission of nearby fluorophores. When a fluorophore is positioned within the intense electromagnetic field of a plasmonic hotspot, such as that formed between closely spaced metallic nanoparticles, both its excitation rate and radiative decay rate can be enhanced, resulting in substantial fluorescence enhancement [47, 48]. The fluorescence enhancement factor (EF) is commonly defined as the ratio of the fluorescence intensity measured in the presence of a plasmonic structure to that measured under a reference condition without the corresponding metallic structure. Because the reference condition and experimental configuration can vary among studies, reported EF values should be interpreted within the context of their respective measurement conditions [49]. However, when a fluorophore is positioned very close to the metal surface, non-radiative energy transfer to the metal can become significant, resulting in fluorescence quenching rather than enhancement. The balance between plasmonic enhancement and metal-induced quenching is system-dependent, and the optimal fluorophore–metal distance should therefore be determined for each PEF system [49, 50]. This distance-dependent behavior highlights the advantage of addressable DNA origami, which enables nanometer-scale control over the spatial organization of fluorophores and plasmonic nanostructures [47, 51, 52]. Such precise spatial organization has enabled DNA origami-based plasmonic nanoantennas to achieve strong fluorescence enhancement and highly sensitive fluorescence detection, including single-molecule detection [23, 47].

Unlike conventional fluorescence strategies that rely on pre-labeled fluorophores and quenchers, de novo fluorescence generation produces fluorescent reporters only after target recognition. Representative examples include the formation of silver nanoclusters (AgNCs) templated by cytosine-rich DNA sequences [53, 54], the self-assembly of copper nanoparticles (CuNPs) on poly(thymine) sequences [55, 56], and fluorogenic RNA aptamers such as Broccoli and Spinach, which generate fluorescence upon binding to specific fluorogens [57, 58]. DNA nanostructures and DNA origami provide an ideal structural framework for these strategies by enabling the precise placement of functional sequences at predefined locations or the programmable exposure and rearrangement of specific DNA motifs in response to target recognition.

Collectively, the integration of these fluorescence signal transduction mechanisms with the structural programmability and precise functional addressability of DNA nanostructures and DNA origami has enabled sophisticated control over fluorescence signal generation, amplification, and readout. As a result, DNA nanotechnology has evolved into a versatile platform for constructing highly sensitive and programmable fluorescence biosensors. In the following sections, representative fluorescence biosensors based on DNA nanostructures and DNA origami are systematically classified and discussed according to their structural designs and sensing mechanisms.

## DNA nanostructure-based fluorescence biosensors

Advances in DNA nanotechnology have enabled the development of a wide variety of DNA nanostructures with precisely controlled geometries and dimensions [59]. These nanostructures are self-assembled through Watson–Crick base pairing and can be rationally designed with nanometer-scale precision. In addition, their highly programmable architectures allow the site-specific incorporation of functional nucleic acids, fluorophores, and nanomaterials, making them versatile platforms for a broad range of biosensing applications [60, 61]. Representative DNA nanostructures include TWJ DNA nanostructures [62], dendritic DNA nanostructures [63], TDNs [64], DNA hydrogels [65], DNA nanoflowers (DNFs) [66], Owing to their distinct structural characteristics, these nanostructures offer unique advantages in signal generation, signal amplification, spatial organization of functional probes, and intracellular delivery. Furthermore, their excellent biocompatibility and efficient cellular uptake have facilitated widespread applications in intracellular biomarker detection and bioimaging [60, 67]. This section classifies representative DNA nanostructures according to their structural features and highlights how each architecture contributes to enhanced fluorescence biosensing through improved signal generation, signal amplification, and biomarker detection.

### TWJ DNA nanostructure-based fluorescence biosensors

TWJ DNA nanostructures have attracted considerable attention as versatile fluorescence biosensing platforms owing to their programmable branched architecture and their ability to precisely organize functional DNA components within confined nanoscale spaces [68]. Unlike conventional linear DNA probes, TWJ structures consist of three independently programmable branches, each of which can be functionalized with target-recognition sequences, fluorescent reporters, signal amplification modules, or sequences for nanomaterial formation [13]. Consequently, TWJs function not merely as structural scaffolds but also as active reaction platforms that convert molecular recognition events into quantitative fluorescence signals.

Early TWJ-based fluorescence biosensors exploited the formation and dissociation of the TWJ itself as a label-free fluorescence signaling mechanism. For the detection of tobramycin (TOB), a target-responsive TWJ was constructed using two TOB-specific aptamers (T1 and T2) and one complementary strand [69]. In the absence of TOB, the three DNA strands assembled into a stable TWJ containing multiple double-stranded DNA regions, allowing PicoGreen (PG) to intercalate and generate strong fluorescence. Upon the addition of TOB, however, the aptamers preferentially bound to the target rather than maintaining the TWJ architecture, leading to partial disassembly of the junction and a reduction in the double-stranded DNA regions. Consequently, PG intercalation decreased, resulting in a proportional reduction in fluorescence intensity. By directly coupling TWJ assembly and disassembly with fluorescence output, this strategy enabled quantitative detection of TOB without the need for fluorescent labeling.

Subsequent studies extended TWJs beyond simple conformational sensing into programmable fluorescence signal amplification platforms. A representative example is the proximity-localized catalytic hairpin assembly (plCHA) system based on a TWJ architecture for nucleic acid detection [69]. In this design, two hairpin probes (H1 and H2) were spatially confined on a TWJ scaffold, thereby overcoming the diffusion limitations associated with conventional solution-phase catalytic hairpin assembly (CHA). The increased local concentration of the hairpins within the TWJ significantly accelerated the CHA reaction, while efficient hairpin opening occurred only in the presence of the target DNA. The reconstructed DNA template subsequently directed the formation of AgNCs, producing a strong turn-on fluorescence signal. This work demonstrated that TWJs can simultaneously function as localized reaction scaffolds for signal amplification and as platforms for fluorescence reporter generation, resulting in rapid reaction kinetics, low background signals, and excellent quantitative analytical performance.

TWJs have also been employed as spatial regulators of the fluorescence properties of preformed nanoclusters. In a three-way DNA device (3WDD), AgNCs were incorporated into hairpin loops, and their fluorescence was modulated through confined strand displacement and transient concatenate ligation reactions [70]. Each branch of the TWJ was functionalized with an AgNC-containing reporter hairpin, a strand-displacement sequence, or a ligation sequence. Upon target recognition, sequential strand displacement and transient ligation reactions occurred within the confined TWJ environment, inducing structural rearrangements around the AgNCs and thereby altering their local fluorescence environment. Unlike conventional fluorescence amplification strategies that generate new fluorophores, this approach directly modulated the emission properties of pre-existing AgNCs to amplify fluorescence signals.

Beyond regulating individual fluorescent nanoclusters, TWJs have been further developed into platforms for spatially organizing multiple fluorescent elements to enable ratiometric fluorescence analysis. In multicluster TWJ DNA nanostructures, AgNC-forming sequences were introduced into each branch, allowing multiple AgNCs to be assembled within a single DNA construct [71]. The branched architecture promoted the ordered arrangement of multiple AgNCs, resulting in enhanced fluorescence intensity while enabling ratiometric fluorescence measurements based on distinct emission wavelengths. By integrating the spatial organization of AgNC arrays with target-induced signal amplification, this strategy achieved high fluorescence responsiveness, excellent reproducibility, and improved sensitivity for quantitative nucleic acid detection.

**Fig. 1:**
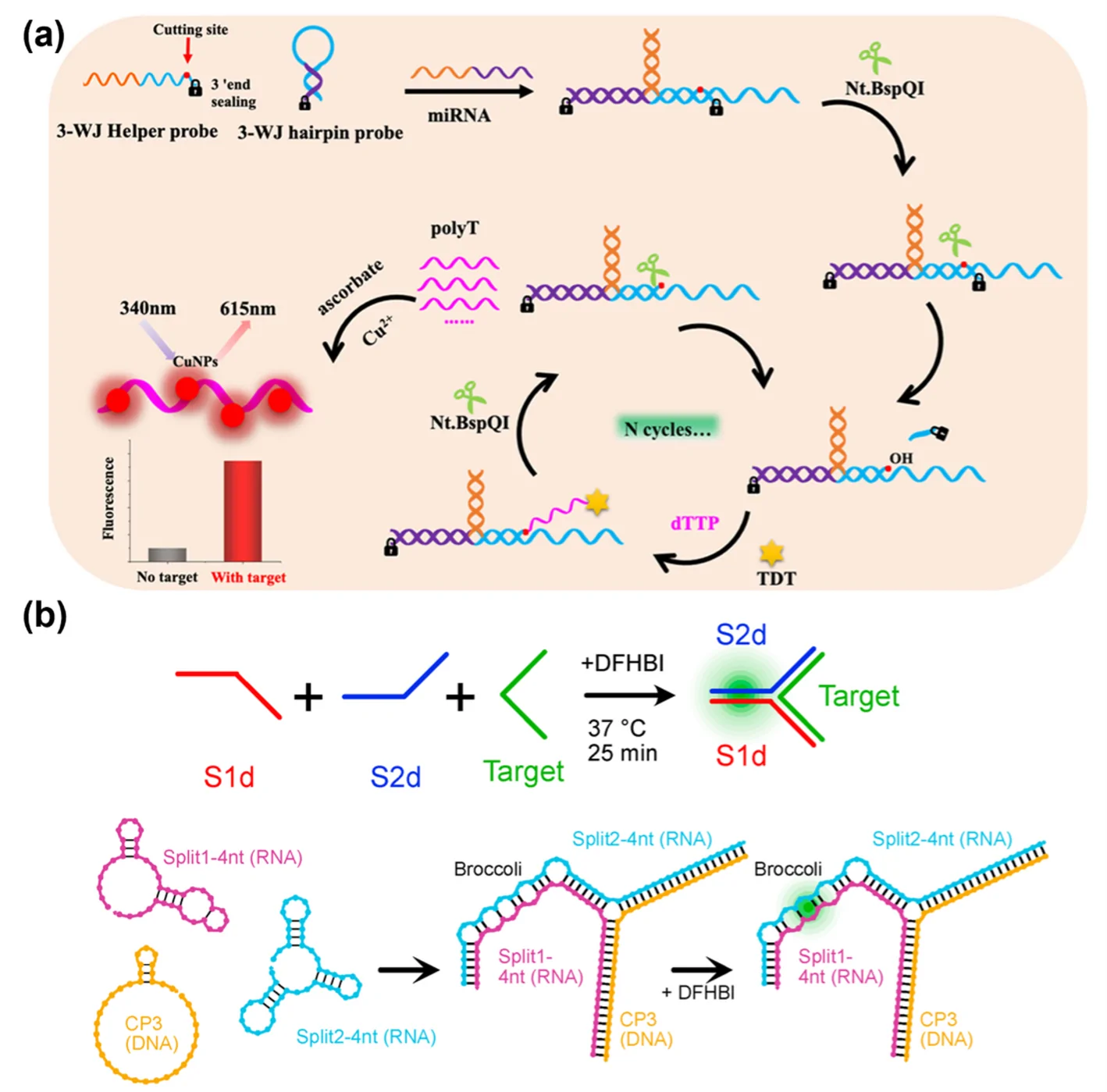
Representative applications of TWJ DNA nanostructures in fluorescent biosensing. (**a**) Schematic illustration of a TWJ DNA nanostructure serving as a target-responsive platform for enzyme-assisted signal amplification in the fluorescence detection of miR-153. Reprinted with permission from [62]. Copyright 2020, American Chemical Society. (**b**) TWJ DNA nanostructure serving as a spatial scaffold for the assembly of a split Broccoli aptamer by positioning its two fragments on separate branches. Reproduced from Ref. [73]. Licensed under CC BY 4.0

TWJs have also been utilized as target-responsive platforms for activating enzyme-assisted signal amplification. To quantitatively detect miRNA-153-3p (miR-153-3p), a fluorescence biosensor combining target-induced TWJ formation with dual enzyme-mediated cascade amplification was developed [62]. Target miRNA initiated the interaction between a hairpin probe and helper DNA, leading to the formation of a stable TWJ, which subsequently activated an amplification reaction involving terminal deoxynucleotidyl transferase (TdT) and the nicking endonuclease Nt.BspQI. This process generated abundant poly(thymine) sequences that served as templates for the self-assembly of fluorescent CuNPs. The method was further evaluated using blood samples from three healthy individuals and five patients with Parkinson’s disease, from which miRNAs were extracted and miR-153-3p was successfully quantified. The results revealed upregulated miR-153-3p expression in patients with Parkinson’s disease, demonstrating the applicability of the method to clinical blood samples. Through the combination of TWJ-mediated structural activation and enzyme-assisted fluorescence amplification, the sensor achieved a detection limit of 1 fM for miR-153-3p, demonstrating the potential of TWJs as programmable amplification platforms for ultrasensitive fluorescence biosensing (Fig. 1a).

More recently, TWJs have been employed as spatial scaffolds for assembling fluorescent reporters. In one strategy, two fragments of a split Broccoli aptamer were positioned at different branches of a TWJ such that target-induced TWJ assembly precisely aligned the fragments to reconstruct the functional Broccoli aptamer [72]. The reconstituted aptamer subsequently bound the fluorogen DFHBI-1T, producing a fluorescence signal whose intensity increased proportionally with the target DNA concentration. This platform enabled quantitative detection of *Campylobacter* spp.-derived DNA at the fM level, demonstrating that TWJs can serve not only as structural scaffolds but also as active sensing platforms that directly couple target recognition with fluorescence generation (Fig. 1b).

Collectively, TWJ DNA nanostructures have evolved from simple conformational fluorescence sensors into multifunctional platforms capable of integrating diverse fluorescence generation and signal amplification strategies. Their programmable branched architecture enables the precise nanoscale organization of recognition elements, fluorescent reporters, and amplification modules, facilitating a wide range of sensing mechanisms, including label-free fluorescence switching, fluorescence generation using AgNCs and CuNPs, ratiometric fluorescence analysis, and enzyme-assisted signal amplification. These unique structural and functional characteristics establish TWJ DNA nanostructures as a promising foundation for next-generation ultrasensitive and quantitative fluorescence biosensing platforms. The analytical characteristics of representative fluorescent biosensing platforms incorporating TWJ DNA nanostructrues, including target analytes, detection mechanisms, signal amplification strategies, LODs, assay times, and validation levels, are summarized in Table 1. 

**Table 1:** Comparison of TWJ-based fluorescence biosensing platforms

Target	Mechanism	Amplification used	LOD	Assay time	Validation level	Ref.
TOB	Target-induced 3WJ structural change alters dsDNA regions	-	21.86 nM	30 min	Human serum	[69]
SARS-CoV-2-related DNA segment (s_2_DNA)	Target-induced 3WDD conformational switching and target recycling, followed by head-to-tail DNA assembly	Target-triggered recycling	0.5 pM	-	10-fold diluted human serum	[70]
Target DNA (T*)	Target-triggered TWJDC assembly with target recycling and multi-AgNC formation	Target-triggered recycling	1.9 pM	-	Human serum	[71]
miR-153-3p	Target-induced 3-WJ formation activates TDT/Nt.BspQI cycling, generating poly-T for fluorescent CuNP self-assembly	Signal amplification induced by the cyclic action of TDT and Nt.BspQI	1 fM	1.75 h	Whole-blood RNA extraction from Parkinson patients and normal individuals	[62]
*Campylobacter spp.* DNA	Target-induced assembly of split Broccoli aptamer, activating fluorescence	-	0.074 ng/µL	~ 20 min	-	[72]

### Hyperbranched and dendritic DNA nanostructure-based fluorescence biosensors

Hyperbranched and dendritic DNA nanostructures have emerged as versatile signal-amplifying platforms for fluorescence biosensing owing to their highly branched architectures, which provide abundant sites for the spatial organization of functional DNA components and fluorescent reporters. Unlike linear DNA probes, these branched nanostructures possess a high probe-loading capacity, allowing a single target-recognition event to initiate multiple fluorescence-generation events and thereby significantly improving detection sensitivity [73]. Furthermore, the close spatial arrangement of reaction sequences and fluorescent probes within the branched framework creates a nanoconfined reaction environment that enhances hybridization efficiency and reaction kinetics [40]. These structural advantages also facilitate integration with diverse nucleic acid amplification strategies, including rolling circle amplification (RCA), hybridization chain reaction (HCR), DNAzyme circuits, and CRISPR-based signal amplification, enabling highly sensitive and quantitative fluorescence biosensing.

**Fig. 2:**
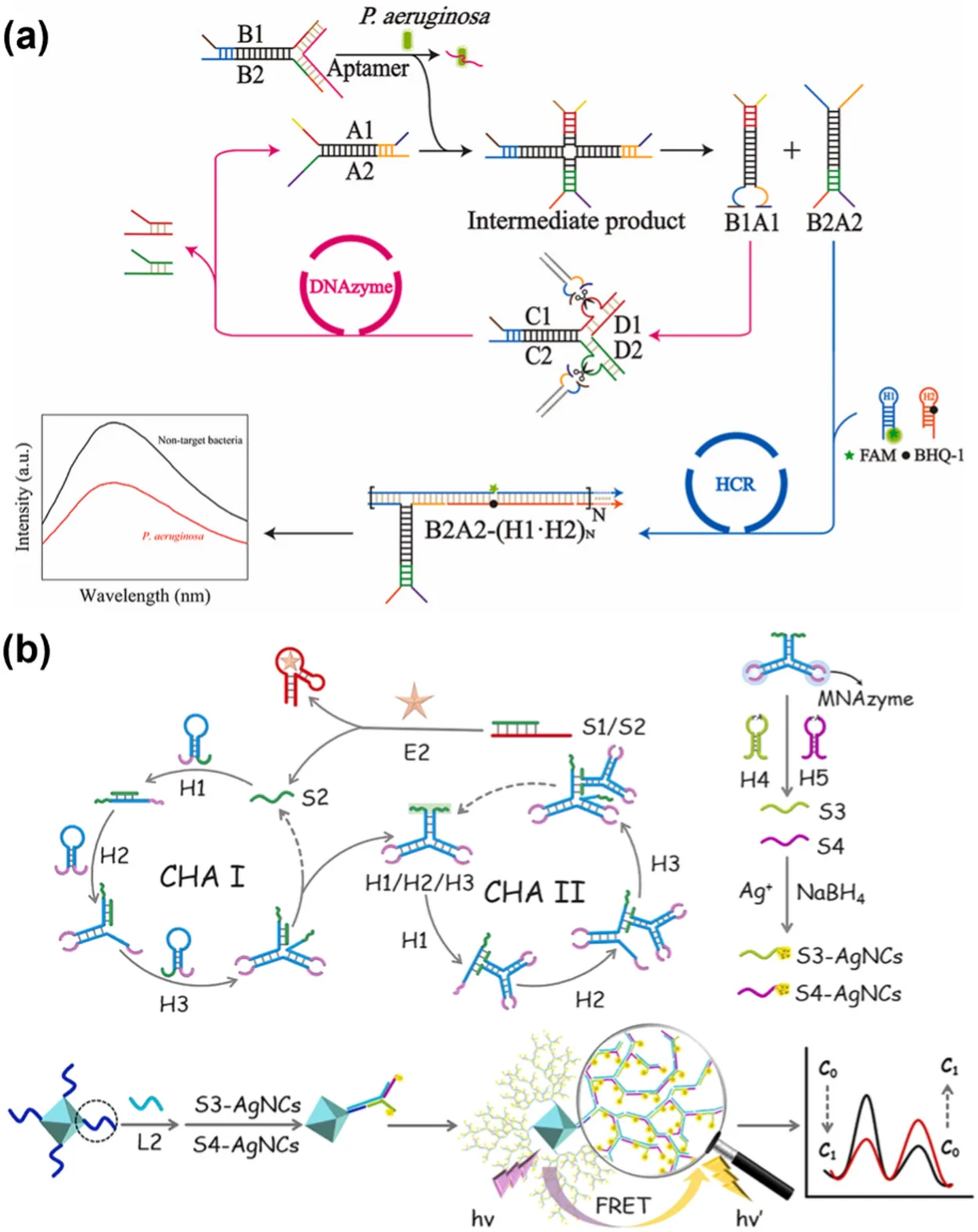
Representative applications of hyperbranched and dendritic DNA nanostructures in fluorescent biosensing. (**a**) Schematic illustration of a hierarchical dendritic DNA network generated by double-stranded DNA branch migration, HCR, and DNAzyme feedback amplification. Reproduced from Ref [74]. Copyright 2022, Elsevier. (**b**) Schematic illustration of hyperbranched DNA as a scaffold for the spatial organization of AgNCs in a dual-emission ratiometric fluorescence sensing platform. Reproduced from Ref [76]. Copyright 2026, Elsevier

Early studies employed DNA ligase to assemble repeatedly branched dendritic DNA architectures that served as fluorescence signal amplification scaffolds. The resulting dendritic nanostructures provided numerous binding sites for fluorescent probes, enabling highly efficient signal accumulation and the ultrasensitive detection of DNA methylation biomarkers at extremely low concentrations [63]. Subsequent studies shifted from conventionally synthesized dendrimers toward amplification-driven strategies that generated dendritic DNA architectures through nucleic acid amplification reactions. Although these amplification products are not structurally identical to classical dendrimers, their repetitive branching topology provides multiple signal-generation sites, resulting in comparable amplification performance. A representative example combined double-stranded DNA branch migration, HCR, and a DNAzyme feedback circuit within a single fluorescence biosensing platform [74]. Upon target recognition, branch migration and HCR sequentially generated a hierarchical dendritic DNA network, which subsequently activated the DNAzyme feedback circuit for continuous signal amplification. This integrated amplification strategy enabled the highly sensitive and quantitative detection of *Pseudomonas aeruginosa* (Fig. 2a).

Hyperbranched DNA has likewise become an important platform for fluorescence signal amplification. Dendritic DNA nanostructures generated through hyperbranched RCA have been successfully incorporated into ratiometric fluorescence biosensors. The highly branched DNA framework produced by hyperbranched RCA provides abundant binding sites for fluorescent probes while increasing their local concentration, thereby improving hybridization efficiency through nanoconfinement [40]. Furthermore, by incorporating a ratiometric detection strategy that measures the ratio between two fluorescence signals upon target recognition, the system minimizes measurement errors arising from environmental fluctuations while simultaneously achieving high analytical accuracy and sensitivity.

Branched DNA nanostructures have also been integrated into CRISPR-based fluorescence biosensors. In one representative study, DNA nanotrees assembled on gold nanomagnetic beads were employed as fluorescence signal amplification scaffolds in combination with CRISPR-Cas12a and the amplification refractory mutation system (ARMS) to detect the *FecB* single-nucleotide mutation with high sensitivity [75]. The DNA nanotree architecture accommodated multiple fluorescent reporters simultaneously, thereby increasing the number of reporter molecules cleaved by activated Cas12a and significantly enhancing the sensitivity of CRISPR-based fluorescence detection.

Recently, hyperbranched DNA has also been employed as a three-dimensional scaffold for the high-density organization of fluorescent nanoclusters. In a representative strategy for detecting the estrogenic environmental pollutant 17β-estradiol (E2), a hyperbranched DNA network was generated through successive aptamer-based nucleic acid amplification reactions, followed by the spatial organization of AgNCs along the branched DNA framework [76]. Simultaneously, a metal–aggregation-induced emission luminogen (metal-AIEgen) framework was introduced to construct a dual-emission ratiometric fluorescence sensing platform based on FRET between AgNCs and the metal-AIEgen. The hyperbranched DNA not only provided abundant nucleation sites for AgNC formation but also promoted uniform AgNC growth and stable fluorescence generation through a nanoconfinement effect that increased the local concentration of template sequences (Fig. 2b).

Collectively, hyperbranched and dendritic DNA nanostructures provide three-dimensional signal amplification scaffolds capable of densely organizing functional DNA components and fluorescent reporters, thereby markedly improving the sensitivity and quantitative performance of fluorescence biosensors. By integrating with diverse nucleic acid amplification strategies, including RCA, HCR, DNAzyme circuits, and CRISPR systems, these branched DNA architectures have evolved beyond passive structural scaffolds into programmable and active signal amplification platforms. Their exceptional signal amplification capability and structural versatility make them highly promising building blocks for the development of next-generation ultrasensitive and multiplexed fluorescence biosensors. The analytical characteristics of representative fluorescent biosensing platforms incorporating hyperbranched and dendritic nanostructures, including target analytes, detection mechanisms, signal amplification strategies, LODs, assay times, and validation levels, are summarized in Table 2. 

**Table 2:** Comparison of hyperbranched and dendritic DNA nanostructure-based fluorescence biosensing platforms

Target	Mechanism	Amplification used	LOD	Assay time	Validation level	Ref.
DNA methylation	Methylation-dependent LDR triggers dendritic DNA assembly, amplifying the fluorescence signal	CHA	0.4 fM	~ 2 h	Human serum	[63]
*Pseudomonas aeruginosa*	Target-induced formation of two DNA Y-junction structures through double-stranded DNA branch migration for signal amplification	HCR and DNAzyme feedback circuit	37 CFU/mL	-	10 times diluted fresh urine samples	[74]
Kanamycin	Target-induced hyperbranched RCA and MNAzyme formation for dual signal amplification	Exo III-assisted recycling amplification and RCA	0.53 fg/mL	80 min	Milk sample and local environmental water sample	[40]
*FecB* mutation in BMPR1B gene	Mutation-specific ARMS amplification followed by Cas12a trans-cleavage and DNA nanotree-mediated fluorescence amplification	Signal amplification using gold nanomagnetic beads	0.001 µM	1 min	Animal sample validation	[75]
E2	E2-triggered dual CHA and MNAzyme-mediated cleavage for AgNC-decorated hyperbranched DNA assembly	CHA	1.2 fg/mL	70 min	Milk, local lake water and soil extract samples	[76]

### TDN-based fluorescence biosensors

TDNs are among the most representative three-dimensional DNA nanostructures and are typically assembled through the self-assembly of four single-stranded DNA molecules with complementary sequences. Since the first DNA tetrahedron was constructed by thermal annealing in 2004 [77], TDNs have been extensively employed in a wide range of biosensing platforms owing to their excellent structural stability and highly programmable architecture. DNA tetrahedra possesses high mechanical rigidity and structural robustness [78], while maintaining functional DNA probes in an upright and well-defined orientation, thereby reducing steric hindrance and improving hybridization efficiency [79]. Moreover, their low cytotoxicity and efficient cellular uptake have enabled broad applications in intracellular biomarker detection and biomolecular delivery. These unique structural characteristics allow TDNs to be readily integrated with various signal generation and signal amplification strategies, making them one of the most versatile platforms for enhancing the performance of optical biosensors. This section highlights representative optical biosensing platforms based on TDNs, with particular emphasis on their roles in signal generation and signal amplification.

**Fig. 3:**
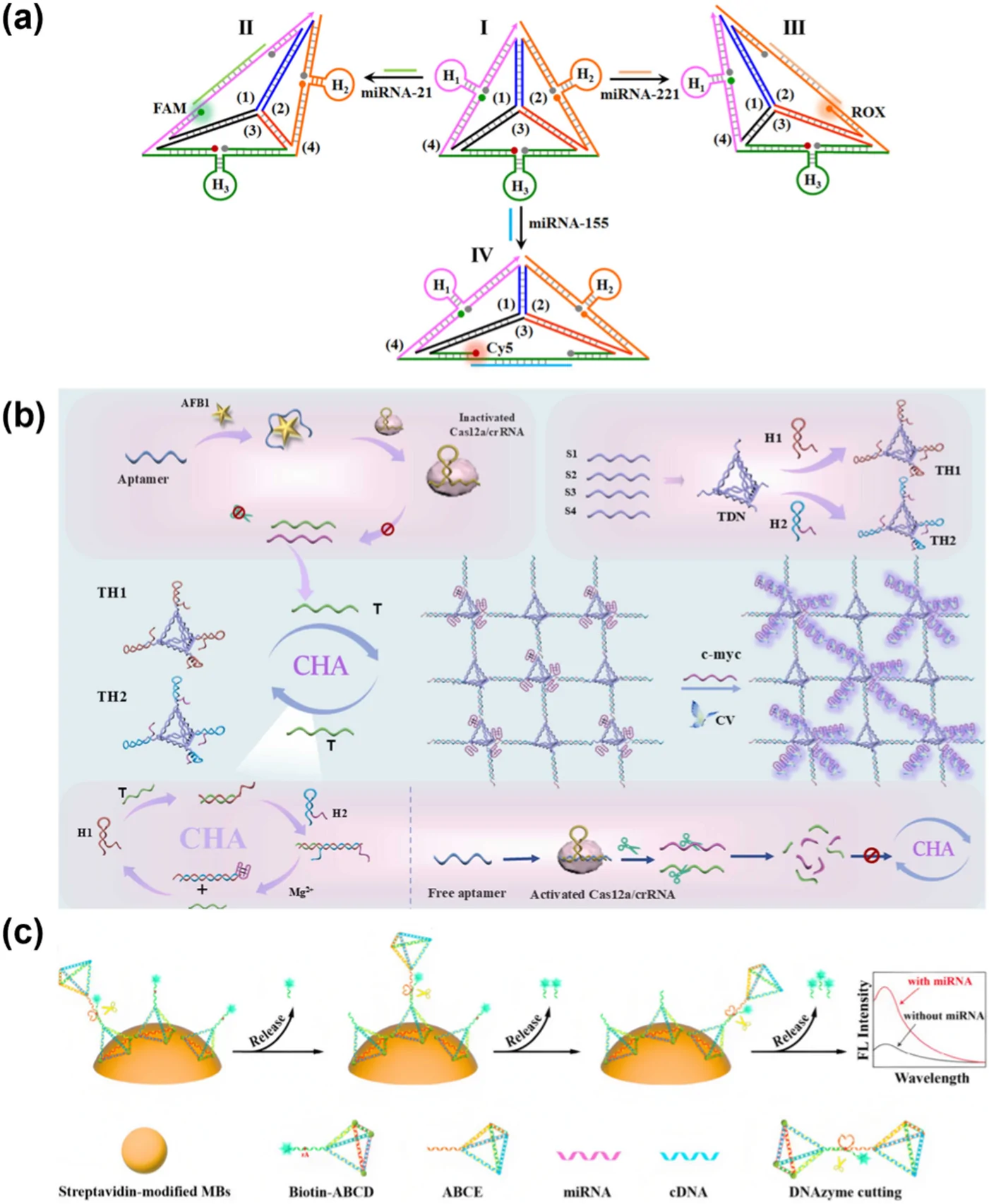
Representative applications of TDNs in fluorescent biosensing. (**a**) Schematic illustration of a multiplexed sensing platform that enables the simultaneous detection of different types of targets by modulating the distance between fluorophore/quencher pairs through selective edge functionalization of a TDN. Reproduced from Ref [64]. Licensed under CC-BY 4.0. (**b**) Illustration of a TDN-assisted CHA system for fluorescence signal amplification. Reproduced from Ref [81]. Copyright 2025, Elsevier. (**c**) Schematic illustration of a TDN-based strategy for alleviating steric hindrance by controlling the spacing between DNA probes immobilized on magnetic beads (MBs). Reprinted with permission from Ref. [67]. Copyright 2026, American Chemical Society

TDNs are relatively easy to fabricate and offer a high degree of structural programmability, allowing precise control over the lengths of their edges and the functionalization of their vertices. This design flexibility enables the site-specific organization of signaling molecules and nanomaterials, making TDNs attractive platforms for multiplexed biosensing and optical signal generation. A multiplexed sensing platform capable of simultaneously detecting three different targets was developed by independently functionalizing the three edges of a DNA tetrahedron [64]. Each edge was modified with a hairpin probe complementary to a specific miRNA (miR-21, miR-221, or miR-155), while distinct fluorophore/quencher pairs were incorporated into the stem region of each hairpin. Upon target recognition, the hairpin structure opened, increasing the distance between the fluorophore and quencher and thereby restoring fluorescence emissions. This design enabled the simultaneous and interference-free detection of three miRNA targets and was further applied to intracellular multiplexed miRNA imaging. Moreover, by modifying the edge design, the same strategy was successfully extended to the simultaneous detection of three endonucleases (EcoRI, Nt.BbvCI, and HindIII) and three protein targets (ATP, VEGF, and thrombin) (Fig. 3a). In another example, only one of the six edges of a DNA tetrahedron was functionalized with a sensing probe for intracellular imaging of the Ago2/miR-21 complex [80]. The functional edge incorporated a DNA/AgNC and a G-quadruplex (G4) as a PET pair, with the two components maintained in close proximity by a hairpin structure. Upon recognition of the Ago2/miR-21 complex, hairpin opening spatially separated the AgNC and G4, suppressing PET and restoring AgNC fluorescence under 560 nm excitation. This strategy demonstrated that the precise spatial organization provided by TDNs enables the selective intracellular imaging of specific nucleic acid complexes.

Compared with conventional single-stranded nucleic acid probes, TDNs provide a greater number of functionalization sites at the same probe concentration while precisely controlling the spatial arrangement of functional probes. These structural advantages reduce steric hindrance, increase the local concentration of functional molecules, and facilitate more efficient nucleic acid amplification reactions. A CRISPR/Cas12a-assisted aptasensor incorporating TDN-acceleratedCHA was developed for the sensitive detection of aflatoxin B1 (AFB1), a representative mycotoxin [81]. CHA hairpin probes (H1 and H2) were immobilized at the four vertices of the DNA tetrahedron. In the presence of AFB1, target binding to the aptamer inhibited Cas12a activation, allowing the trigger strand to initiate the CHA reaction. The alternating opening of H1 and H2 subsequently induced the assembly of TDN clusters containing G4 sequences. Upon the addition of K^+^ ions, G4 structures were formed and subsequently bound crystal violet (CV) to generate fluorescent G4/CV complexes (Fig. 3b). Compared with conventional free H1 and H2 probes, immobilization of the CHA probes on the TDN scaffold shortened the inter-probe distance and increased their local concentration, thereby significantly accelerating CHA kinetics. A similar strategy was employed in a TDN-DNAzyme-based CHA reaction (TDCR) for the stable monitoring of intracellular miRNA expression in living cells [82]. Target-activated DNAzymes initiated the CHA reaction, resulting in the aggregation of H1/H2-functionalized TDNs and the generation of amplified fluorescence signals. The platform achieved an excellent limit of detection (LOD) of 1.32 fM, demonstrating its high analytical sensitivity. Similarly, a TDN-based CHA strategy was also applied to the sensitive intracellular imaging of miR-21 [83], further demonstrating that the well-defined spatial organization provided by TDNs can substantially enhance the efficiency of nucleic acid amplification reactions. The study demonstrated that the sensor remained stable and functional in 10% human serum for several hours, confirming its durability, while cytotoxicity tests in MCF-7 and HEK-293T cells demonstrated its low cytotoxicity. Furthermore, miR-21 was successfully detected in serum samples collected from 15 patients with colorectal cancer and 10 healthy donors, achieving 100% sensitivity and specificity. Although the cohort size was relatively small, these results highlight the clinical potential of the TDN-based miRNA detection platform.

Beyond nucleic acid amplification, TDNs have also been utilized for enzyme activity sensing by optimizing the spatial arrangement of functional probes. A fluorescence platform was developed for detecting the activity of uracil DNA glycosylase (UDG), a key enzyme involved in the base excision repair (BER) pathway, by incorporating uracil-containing nucleic acid probes into a TDN scaffold [79]. In the presence of UDG, T-CUNPs were generated to produce fluorescence signals, enabling sensitive detection of UDG in both serum samples and cancer cells. Furthermore, compared with conventional single- or double-stranded DNA probes, the TDN-based platform provided a greater number of recognition sites at the same probe concentration, achieving an ultralow LOD of 8.6 × 10^− 5^ U/mL. TDNs have also been employed to improve the cleavage kinetics of conventional fluorescence-quencher reporters used in CRISPR/Cas12a trans-cleavage assays. An DNAzyme-embedded framework nucleic acid (FNAzyme) platform was constructed by encoding the CLICK-17 DNAzyme within a TDN scaffold [61]. When applied to the detection of methicillin-resistant *Staphylococcus aureus* (MRSA), the increased local density of DNAzymes on the TDN significantly enhanced the trans-cleavage activity of Cas12a, resulting in sensitive detection with an excellent LOD of 18 CFU/mL. Another strategy utilized TDNs to alleviate steric hindrance arising from the high local density of DNA probes immobilized on solid surfaces in nanoparticle-based nucleic acid sensing systems [67]. In this design, one type of TDN was conjugated onto magnetic beads to precisely control the spacing between DNA probes, whereas a second TDN was engineered to function as a DNAzyme. DNAzyme-mediated cleavage subsequently released fluorescently labeled substrate strands into the supernatant, enabling the sensitive detection of miR-205 with an LOD of 5.8 pM (Fig. 3c).

Overall, TDNs have emerged as versatile DNA nanostructures capable of precisely organizing signaling molecules, increasing the local concentration of functional probes, and integrating efficiently with a wide range of signal amplification strategies owing to their excellent structural stability and highly programmable architecture. Their excellent biocompatibility and efficient cellular uptake have also enabled broad applications in intracellular biomarker detection and bioimaging. Furthermore, several studies have demonstrated the detection performance of TDN-based biosensors using serum samples, highlighting their considerable potential for advancing molecular diagnostic applications. These characteristics enable TDN-based fluorescence biosensors to achieve high analytical performance while providing valuable design principles for the development of next-generation functional DNA nanostructures. The analytical characteristics of representative fluorescent biosensing platforms incorporating TDNs, including target analytes, detection mechanisms, signal amplification strategies, LODs, assay times, and validation levels, are summarized in Table 3. 

**Table 3:** Comparison of TDN-based fluorescence biosensing platforms

Target	Mechanism	Amplification used	LOD	Assay time	Validation level	Ref.
miRNA (miR-21, miR-221, and miR-155), endonucleases (EcoRI, Nt.BbvCI, and HindIII), protein (ATP, VEGF, and thrombin)	Target-induced structural reconfiguration	-	1 nM (miR-21 and miR-155)	-	Cellular imaging	[64]
Ago2/miR-21 complex, RNase H	Target-induced distance modulation between G4 and AgNC motifs, restoring fluorescence	-	4.54 nM	30 min	Cell lysates, cellular imaging	[80]
miR-155	DNAzyme-mediated substrate cleavage followed by CHA for dual signal amplification	DNAzyme and CHA-mediated signal amplification	1.32 fM	~ 1 h	Cellular imaging	[82]
miR-21	Target-induced assembly of three hairpin-functionalized TDNs with target recycling	CHA	20 pM	2.5 h	10% human serum, cell imaging, peripheral blood from colorectal cancer (CRC) patients and healthy controls (HCs)	[83]
UDG	TDN collapse-induced release of ssDNA for rapid T-CuNP synthesis	-	8.6 × 10^− 5^ U/mL	~ 2.5 h	Cell lysates	[79]
miR-205	target-induced DNAzyme activation and substrate cleavage	DNAzyme-mediated signal amplification	5.8 pM	2 h	10-fold diluted human serum and cell lysates	[67]

### DNA hydrogel-based fluorescence biosensors

DNA hydrogels are three-dimensional hydrated networks formed through complementary DNA hybridization, self-assembly, or nucleic acid amplification reactions. Owing to their high water content, porous architecture, and the inherent sequence specificity and programmability of DNA, these hydrogels provide versatile platforms for the spatial organization of functional DNA components and nanomaterials [65]. Unlike linear DNA probes, DNA hydrogels enable the simultaneous integration of multiple recognition elements and fluorescent reporters within a single three-dimensional matrix, allowing target recognition, fluorescence activation, signal amplification, and target enrichment to be achieved in one platform [84]. Furthermore, the confined microenvironment within the hydrogel generates a nanoconfinement effect that increases the local concentration of reactants, thereby enhancing hybridization efficiency and reaction kinetics [20]. Early DNA hydrogel-based fluorescence biosensors primarily generated signals through hydrogel-induced changes in the local microenvironment of fluorescent molecules. More recently, these systems have evolved into multifunctional platforms capable of organizing fluorescent nanomaterials and integrating CRISPR-mediated signal amplification.

**Fig. 4:**
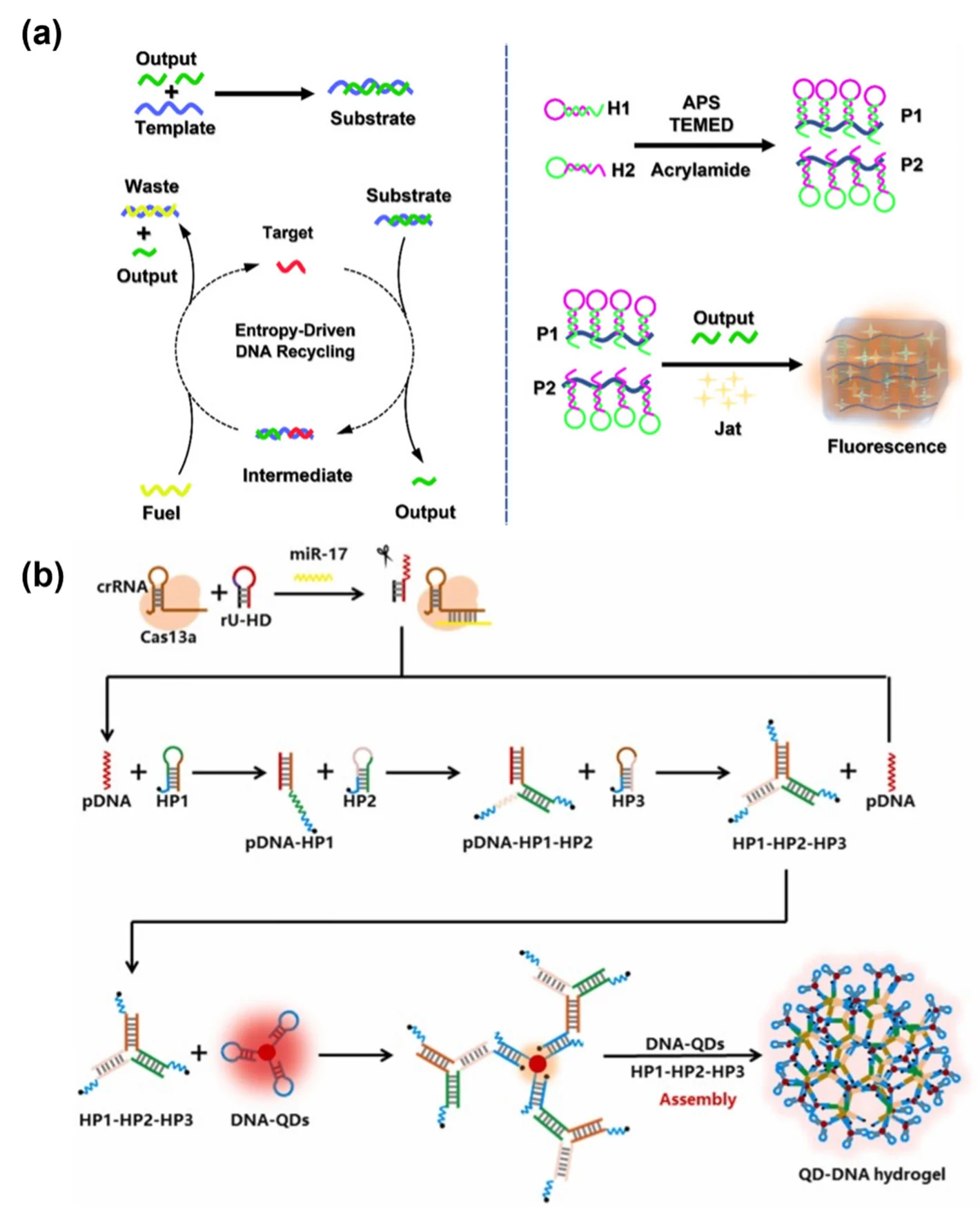
Representative applications of DNA hydrogels in fluorescent biosensing. (**a**) Schematic illustration of a DNA hydrogel-based turn-on fluorescence platform in which an entropy-driven amplification process induces the formation of a fluorescent DNA hydrogel, enabling the spatial confinement of a water-soluble AIEgen. Reprinted with permission from [87]. Copyright 2023, American Chemical Society. (**b**) Schematic illustration of a CRISPR/Cas13a-responsive DNA hydrogel platform in which target miRNA triggers hydrogel assembly and fluorescence amplification through the spatial organization of QD-functionalized DNA components. Reproduced from Ref. [85]. Copyright 2023, Elsevier

A representative example is a DNA hydrogel-based fluorescence sensing platform exploiting aggregation-induced emission (AIE). In this system, target-triggered self-assembly of DNA building blocks produced a three-dimensional hydrogel network, in which the molecular motions of embedded AIE fluorophores were spatially restricted, resulting in strong fluorescence emission [65]. For prostate-specific antigen (PSA) detection, DNA building blocks containing PSA-specific aptamers were designed to undergo target-dependent cross-linking and hydrogel formation. Within the assembled hydrogel, the rotational and vibrational motions of the AIE fluorophores were suppressed, leading to enhanced fluorescence. In contrast, the presence of PSA induced aptamer-target binding, which inhibited hydrogel formation or altered the hydrogel architecture, thereby reducing the fluorescence intensity. This strategy enabled quantitative PSA detection through structure-dependent fluorescence modulation and demonstrated that DNA hydrogels function not only as structural scaffolds but also as active three-dimensional sensing platforms capable of regulating the emission environment of fluorophores. Importantly, the platform was further evaluated using human serum, including patient-derived serum samples, demonstrating its potential for fluorescence-based PSA analysis in clinically relevant samples. Subsequently, label-free DNA hydrogel fluorescence biosensors based on AIE activation were developed. In one representative system, a water-soluble natural AIEgen was incorporated into a DNA hydrogel, where the self-assembled three-dimensional DNA network spatially confined the AIEgen and suppressed its non-radiative molecular motions, thereby producing a strong turn-on fluorescence signal [85]. Because hydrogel formation occurred only in the presence of the target DNA, target recognition could be directly converted into fluorescence output without requiring covalent fluorescent labeling. These results demonstrated that DNA hydrogels are not merely carriers for fluorescent molecules but functional platforms capable of activating fluorescence through precise regulation of the local microenvironment surrounding AIE fluorophores (Fig. 4a).

DNA hydrogels have also been employed as platforms for high-density organization of fluorescent nanoclusters to achieve signal amplification. In a DNA-functionalized hydrogel for miRNA detection, target-recognition sequences and templates for AgNC formation were integrated within the same hydrogel network [20]. Upon target recognition, hybridization or strand displacement reactions exposed cytosine-rich sequences that subsequently templated AgNC formation. The confined three-dimensional hydrogel environment generated a pronounced nanoconfinement effect, concentrating AgNC nucleation sites and promoting uniform nanocluster formation together with stable fluorescence emission. Furthermore, the presence of numerous AgNC-forming sites within a single hydrogel enabled fluorescence responses proportional to the target concentration, thereby allowing quantitative miRNA analysis. A similar concept has been extended to quantum dot (QD)-integrated CRISPR-responsive DNA hydrogels. In a CRISPR/Cas13a-mediated QD-DNA hydrogel platform, target miRNA activated the Cas13a-crRNA complex, whose collateral trans-cleavage activity cleaved the uracil ribonucleotide-embedded hairpin DNA and released primer DNA. The released primer DNA subsequently initiated toehold-mediated strand displacement (TMSD) amplification, generating multiple DNA quencher structures that assembled with QD-functionalized DNA components to form a QD-DNA hydrogel [84]. The resulting hydrogel network densely organized QD-functionalized DNA components, while the BHQ-modified DNA quenchers efficiently quenched QD fluorescence. By combining the sequence specificity of CRISPR/Cas13a with TMSD amplification and DNA hydrogel-based signal modulation, this programmable biosensing platform converted miRNA recognition into an amplified fluorescence output (Fig. 4b).

More recently, the structural advantages of DNA hydrogels have been translated into practical analytical platforms. A representative example integrated a DNA hydrogel into a three-dimensionally printed microextractor for the direct fluorescence detection of free antibiotic resistance genes (fARGs) in wastewater [86]. DNA probes immobilized at high density within the hydrogel enabled efficient target capture in complex sample matrices. Upon target recognition, strand displacement amplification (SDA) generated amplified DNA products, which induced DNA hydrogel contraction and promoted the aggregation of tetraphenylethene (TPE)-based aggregation-induced emission (AIE) fluorophores, resulting in enhanced fluorescence signals. The combination of the microextractor and DNA hydrogel enabled sample enrichment, target concentration, SDA-based signal amplification, and fluorescence detection within a single integrated platform, demonstrating the practical potential of DNA hydrogel-based biosensors for environmental analysis. The platform was further validated using aquaculture water, fish samples, and environmental water, demonstrating its potential for direct analysis of antibiotic resistance genes in complex environmental samples.

Collectively, DNA hydrogel-based fluorescence biosensors have evolved from early systems that relied on hydrogel-induced modulation of fluorophore microenvironments to sophisticated platforms incorporating AIE activation, fluorescence amplification using AgNCs and QDs, CRISPR-responsive signal generation, and practical analytical devices for real-world sample analysis. Importantly, validation using patient-derived serum, biologically relevant samples, and complex environmental matrices highlights the expanding potential of DNA hydrogels for translational and real-world fluorescence biosensing applications. The three-dimensional hydrogel network provides an ideal framework for the high-density organization of functional DNA components and fluorescent reporters, while the nanoconfinement effect enhances both reaction efficiency and fluorescence output. These unique structural and functional advantages establish DNA hydrogels as highly promising platforms for the development of next-generation ultrasensitive and quantitative fluorescence biosensors. The analytical characteristics of representative fluorescent biosensing platforms incorporating DNA hydrogels, including target analytes, detection mechanisms, signal amplification strategies, LODs, assay times, and validation levels, are summarized in Table 4. 

**Table 4:** Comparison of DNA hydrogel-based fluorescence biosensing platforms

Target	Mechanism	Amplification used	LOD	Assay time	Validation level	Ref.
PSA	AIE-based fluorescence modulation via PSA-induced hydrogel dissolution	AIE-based signal enhancement	4.4 pg/mL	~ 105 min	Human serum, patient serum	[65]
miR-21	AIE-based fluorescence activation	Entropy-driven DNA circuit	0.049 fM	~ 160 min	100-fold diluted human serum	[85]
miR-141	Target-induced AgNC formation and AIE	-	2 pM	> 2 h	Human serum	[20]
miR-17	CRISPR-Cas cleavage, TMSD amplification, and BHQ-mediated QD fluorescence quenching	Cas 13a trans-cleavage and TMSD	182 aM	~ 60 min	100-fold diluted human serum and cell-derived RNA	[84]
fARGs	SDA-generated output DNA induces DNA hydrogel contraction and TPE-based aggregation for fluorescence enhancement	SDA	0.165 fM (bla-TEM), 0.231 fM (tetM), 0.171 fM (sul-1), 0.159 fM (bla-CTX-M-1)	~ 40 min	Aquaculture water, fish and environmental water	[86]

### Other Spatially Organized Scaffolds-based Fluorescence Biosensors

In addition to the representative DNA nanostructures described above, a variety of other spatially organized scaffolds have also been developed as fluorescence biosensing platforms [21]. Although these systems are difficult to classify into a single structural category, they share a common design principle of utilizing programmable DNA scaffolds to spatially organize functional molecules and regulate fluorescence signaling. The following section highlights representative fluorescence biosensing platforms based on these diverse DNA scaffold architectures.

Early studies primarily focused on regulating the spatial organization and emission properties of fluorophores using DNA scaffolds. A representative example is a large-area DNA nanoarray assembled through RCA, in which fluorescently labeled DNA strands were periodically organized to precisely control the spacing and interactions between fluorophores [87]. This spatial organization enabled programmable fluorescence switching and signal modulation, demonstrating the utility of DNA scaffolds for controlling photophysical behavior. Subsequent studies expanded this concept by integrating DNA scaffolds with functional nanomaterials. In one representative platform, RCA-generated DNA scaffolds were combined with DNA-templated QD self-assembly to construct fluorescence biosensors for sensitive microbial DNA detection [88]. The three-dimensional DNF produced by RCA provided repetitive DNA sequences that served as high-density scaffolds for organizing fluorescent nanomaterials. Using phosphorothioate-modified DNA templates, CdTe QDs were synthesized in situ within the DNA framework without requiring complicated surface modification procedures. By integrating the amplification capability of RCA with the high quantum yield and excellent photostability of QDs, the DNF-QD platform generated stable fluorescence outputs while demonstrating that DNA scaffolds can function not only as structural supports for fluorophore organization but also as programmable templates for assembling functional nanomaterials.

**Fig. 5:**
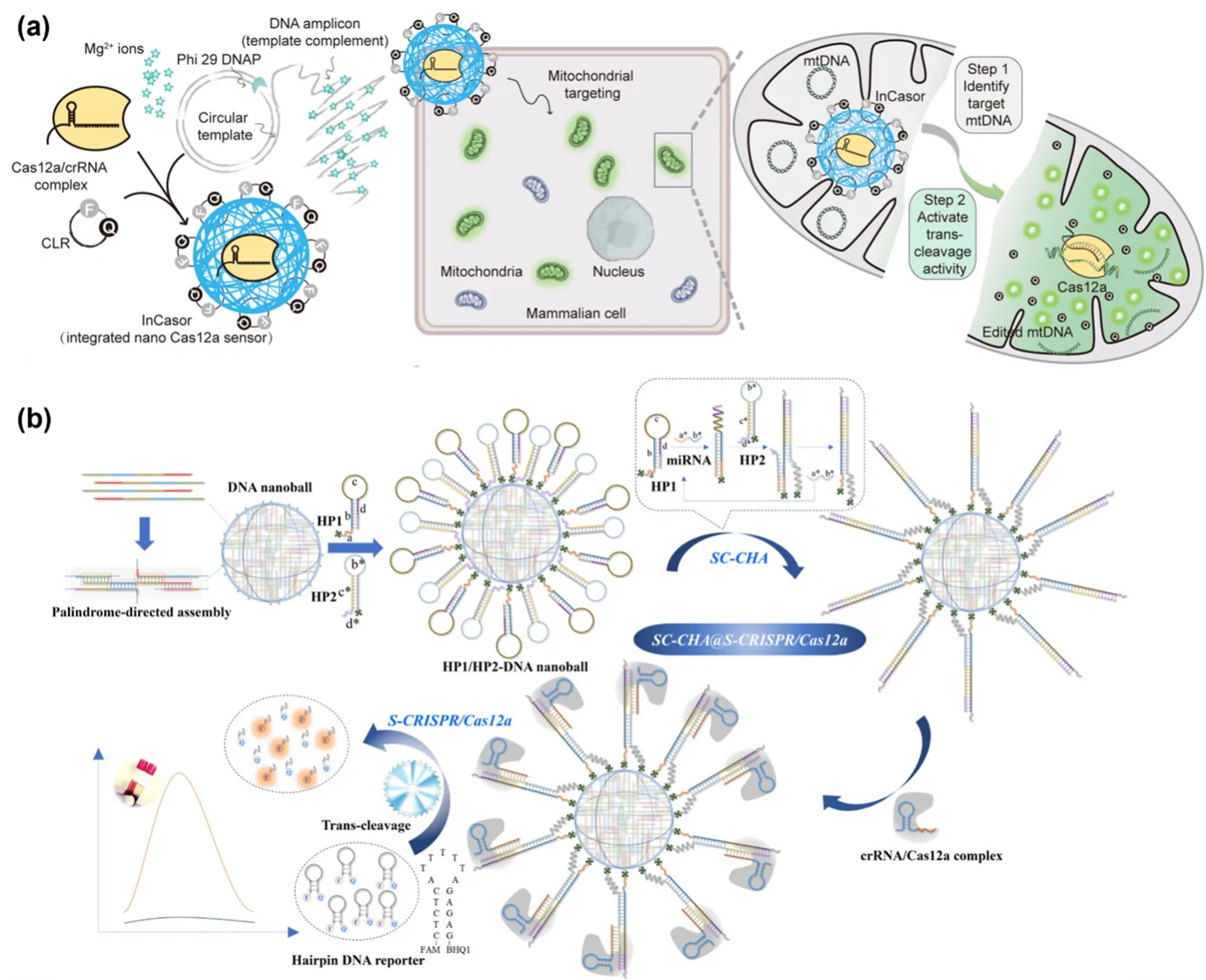
Representative applications of DNF and DNA nanoball in fluorescent biosensing. (**a**) Schematic illustration of a DNF-based InCasor platform integrating Cas12a/crRNA complexes and fluorescent reporters within a DNF scaffold for amplified intracellular imaging of mtDNA mutations. Reproduced from Ref [66]. Licensed under CC BY 4.0. (**b**) Schematic illustration of a spatially confined CRISPR/Cas12a platform integrating CHA-responsive hairpin DNA reporters within a DNA nanoball framework for enhanced fluorescence amplification. Reprinted with permission from [92]. Copyright 2025, American Chemical Society

More recently, DNFs have evolved into multifunctional three-dimensional DNA scaffolds possessing repetitive DNA sequences and exceptionally high loading capacities. These architectures have been further integrated with CRISPR systems to enable intracellular and in vivo imaging of genetic mutations [66]. DNFs simultaneously accommodate multiple functional components—including fluorescent reporters, CRISPR machinery, and targeting ligands—within a single nanostructure, thereby integrating molecular recognition with signal amplification. A representative example is the integrated nano Cas12a sensor (InCasor), which was constructed using RCA-generated DNA/Mg^2+^ hybrid nanoflowers. The nanoflower scaffold spatially organized Cas12a/crRNA complexes, circular fluorescent reporters (CLRs), and cell and mitochondria targeting modules. Following recognition of target mitochondrial DNA (mtDNA) mutations, activated Cas12a generated amplified fluorescence signals through collateral trans-cleavage of the fluorescent reporters, enabling direct intracellular imaging of specific mtDNA mutations (Fig. 5a). The sensing capability was further demonstrated in living cells and tumor-bearing mice, with ex vivo tissue imaging providing additional validation of the platform for intracellular and in vivo molecular imaging.

The application of DNF-based CRISPR sensing has subsequently been extended to disease monitoring in vivo. A renal-clearable CRISPR nanosensor combined DNA nanostructures with Cas12a to enable non-invasive monitoring of tumor progression and metastasis through the detection of mtDNA mutations [89]. Mutation-specific crRNAs, Cas12a, and fluorophore-quencher reporters were spatially organized within a DNA framework to generate fluorescence signals upon target recognition. Owing to its renal-clearable design, the nanosensor enabled urine-based analysis for monitoring tumor progression. Importantly, the nanosensor was validated in mouse tumor models for longitudinal tumor progression and lung metastasis monitoring, demonstrating the potential of DNA nanostructure-based fluorescence sensing for non-invasive in vivo disease monitoring. These studies demonstrate that DNA scaffolds have evolved beyond conventional signal amplification platforms into programmable fluorescence nanosensors capable of intracellular molecular imaging and in vivo disease monitoring.

In parallel, a variety of three-dimensional DNA scaffolds assembled through DNA self-assembly have been developed to spatially organize functional molecules and regulate biomolecular reactions within confined nanoscale environments. These self-assembled DNA architectures provide abundant binding sites and high loading capacities, serving as nanoreactors that spatially confine CRISPR components and enhance intermolecular interactions during signal amplification. For example, a fluorescence biosensor combining a three-dimensional spherical DNA framework with CRISPR-Cas12a for H1N1 virus detection spatially organized multiple target-recognition elements and reaction modules within the DNA framework, thereby improving the accessibility between Cas12a-crRNA complexes and fluorescent reporters and enhancing Cas12a-mediated trans-cleavage for fluorescence signal amplification [90]. The platform was further evaluated using chicken serum, bovine serum, and milk, demonstrating fluorescence detection in diverse complex sample matrices beyond buffer-based assays. Similarly, a spatially confined CRISPR/Cas12a platform incorporated CHA-responsive hairpin DNA reporters into a spherical DNA framework. The confined nanospace increased collision frequency and local reaction efficiency among the reaction components, thereby enhancing cascade signal amplification [91]. This structural confinement effect effectively coupled Cas12a trans-cleavage with catalytic hairpin assembly, enabling highly sensitive nucleic acid fluorescence detection (Fig. 5b).

More recently, the spatial organization of DNA nanostructures has been exploited not only to control the positioning of functional molecules but also to regulate biomolecular reactions by engineering the topology and structural rigidity of the DNA framework itself. A representative example is a de novo-engineered circular DNAzyme platform, in which linear DNAzymes were converted into topologically constrained circular architectures to increase structural rigidity and precisely regulate catalytic activity [21]. By combining DNAzymes with distinct reaction kinetics, temporally encoded fluorescence signatures were generated, enabling multiplex target discrimination while demonstrating that spatially organized DNA nanostructures can precisely regulate both molecular conformational freedom and reaction dynamics.

Collectively, fluorescence biosensors based on other spatially organized scaffolds have evolved from early DNA nanoarrays and RCA-derived DNFs to sophisticated three-dimensional self-assembled DNA frameworks and CRISPR-integrated nanosensing platforms. Through precise control of the spatial arrangement of functional components and local reaction environments within programmable DNA scaffolds, these systems significantly improve fluorescence signal generation, energy transfer efficiency, and biomolecular reaction kinetics. More importantly, other spatially organized scaffolds have progressed beyond passive structural supports to become programmable reaction platforms that simultaneously coordinate molecular recognition, signal amplification, and fluorescence readout, establishing them as key enabling technologies for next-generation ultrasensitive and multifunctional fluorescence biosensors. Notably, validation in serum, food and environmental matrices, as well as intracellular imaging, tumor monitoring, and ex vivo tissue analysis, highlights the expanding applicability of these platforms toward biologically and clinically relevant sensing scenarios. The analytical characteristics of representative fluorescent biosensing platforms incorporating other spatially organized scaffolds, including target analytes, detection mechanisms, signal amplification strategies, LODs, assay times, and validation levels, are summarized in Table 5. 

**Table 5:** Comparison of other spatially organized scaffolds-based fluorescence biosensing platforms

Architecture	Target	Mechanism	Amplification used	LOD	Assay time	Validation level	Ref.
RCA-generated DNFs integrated with DNA-templated CdTe QDs	dsrC gene of sulfate-reducing bacteria	Target-triggered RCA, DNF formation, DNA-templated CdTe QD assembly, and fluorescence generation	RCA and DNF-mediated signal enhanement	1.3 nM	> Overnight	Seawater and diluted human serum	[87]
RCA-generated DNA nanoladder with FAM-labeled thrombin aptamer staples	Thrombin	Target binding to the aptamer and fluorescence activation	RCA	-	-	-	[88]
DNF integrated with Cas12a/crRNA and circular fluorophore-quencher reporter	Mitochondrial DNA (ND4 gene)	Cas12a/crRNA-mediated mtDNA recognition, trans-cleavage of the fluorophore–quencher reporter, and fluorescence activation	Cas12a trans-cleavage and RCA-generated DNF-mediated signal enhancement	~ 1 fM	~ 6 h	Live cell imaging, in vivo tumor imaging and ex vivo tissue imaging	[66]
DNF integrated with Cas12a/crRNA and circular fluorescent reporter	Tumor-specific mitochondrial DNA mutation	Cas12a/crRNA-mediated SNV recognition, Cas12a trans-cleavage of the fluorophore–quencher CLR, fluorescent biomarker generation, extracellular release, renal clearance, and urine fluorescence detection	Cas12a trans-cleavage and RCA-generated DNF-mediated signal enhancement	50 copies of mutant mtDNA in living cells	~ 6 h	Mouse tumor model, urine-based tumor progression monitoring and lung metastasis monitoring	[89]
3D spherical DNA nanostructure integrated with H1N1 aptamer and Cas12a activator	Intact H1N1 virus	H1N1 binding to the aptamer, conformational change, release of the Cas12a activator, Cas12a/crRNA-mediated trans-cleavage of the HEX/BHQ1 reporter, and fluorescence generation	Cas12a trans-cleavage	0.17 copies/µL	~ 40 min	Chicken serum, bovine serum and milk	[90]
DNA nanoball-based spherical CRISPR/Cas12a system with spatially confined CHA	miR-21	miR-21 binding, spatially confined CHA on the DNA nanoball, CHA product-mediated activation of Cas12a/crRNA, Cas12a trans-cleavage of the hairpin DNA reporter, and fluorescence generation	CHA and Cas12a trans-cleavage	13.75 fM	~ 120 min	Human serum	[91]

### Dynamic DNA Nanostructure-based Fluorescence Biosensors

Dynamic DNA nanostructures are active DNA platforms capable of undergoing structural reconfiguration, locomotion, or repetitive molecular motion in response to external stimuli. Unlike static DNA scaffolds, these dynamic architectures convert target recognition events into amplified fluorescence outputs [93]. These are typically integrated with programmable nucleic acid reactions such as strand displacement, CHA, and DNAzymes to generate multiple fluorescence signals from a single target recognition event [92]. These dynamic signal amplification strategies have therefore emerged as powerful fluorescence biosensing platforms for the ultrasensitive quantitative detection of miRNAs, pathogen-derived nucleic acids, and a wide range of biomolecules.

DNA walker-based fluorescence biosensors are generally designed such that the walker strand migrates along a predefined DNA track, sequentially activating multiple fluorescent reporters. Early studies combined a three-dimensional bipedal DNA walker with CHA, in which target-induced walker motion initiated successive hairpin assembly reactions, resulting in cascade fluorescence signal amplification. By integrating dynamic molecular motion with isothermal nucleic acid amplification, these systems enabled highly sensitive quantitative detection of thrombin and miRNAs [93, 94].

Subsequently, DNA walker systems were further integrated with enzyme-mediated amplification and spatially confined reaction environments to improve reaction efficiency and signal amplification performance. An enzyme-assisted 3D DNA walker–CHA platform was developed as a fluorescence biosensor for pathogen-derived nucleic acid detection, where enzyme-triggered walker activation and repeated hairpin assembly generated amplified fluorescence signals [95]. The platform was further evaluated using chicken and milk samples, demonstrating its applicability to the detection of bacterial targets in complex food-related matrices. In another study, a localized CHA-based 3D DNA walker confined both the walker and hairpin amplification modules within a restricted nanospace, thereby increasing the local concentration of reaction components and significantly enhancing reaction kinetics [92]. This spatially confined amplification strategy enabled highly sensitive detection of Zika virus-related nucleic acids, demonstrating that DNA walkers can function not only as molecular motors but also as spatially organized amplification platforms.

Dynamic DNA nanostructures were subsequently combined with AuNPs to further enhance walker mobility and fluorescence signal generation. A single-legged DNA walker assembled on AuNPs utilized the nanoparticle surface to immobilize a high density of DNA probes, coupling target-induced CHA with walker movement to amplify fluorescence signals [96]. The AuNPs provided high probe loading capacity and a stable reaction interface, substantially improving walker-mediated amplification efficiency and enabling highly sensitive detection of prokaryotic pathogens. The sensing performance was further evaluated using bacterial genomic DNA, demonstrating the applicability of the platform to target detection in extracted biological samples.

DNA walker systems have also been extended to aptamer-based small-molecule sensing. A DNA walking machine employing the nicking endonuclease Nb.BbvCI integrated aptamer recognition with enzyme-mediated DNA walker amplification for the detection of deoxynivalenol (DON) [97]. Upon binding of DON to its aptamer, the DNA walker was activated, and repeated nicking by Nb.BbvCI drove continuous walker migration along the DNA track. Consequently, a single target recognition event activated multiple fluorescent reporters, producing a strong amplified fluorescence signal. This strategy successfully expanded DNA walker technology beyond nucleic acid sensing to small-molecule detection while maintaining excellent analytical sensitivity.

**Fig. 6:**
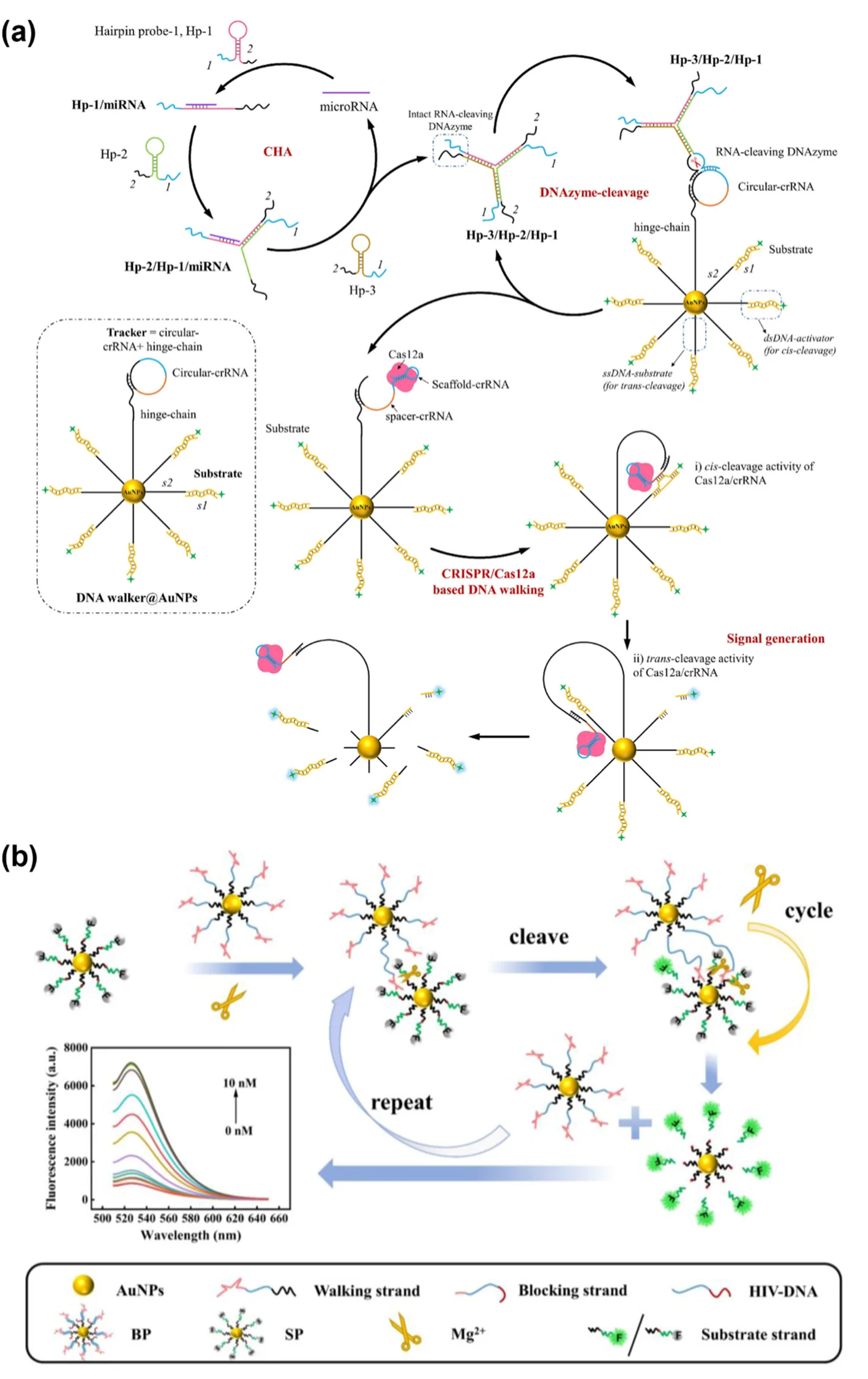
Representative applications of dynamic DNA nanostructures in fluorescent biosensing**.** (**a**) Schematic illustration of a CHA–DNAzyme junction-mediated circular crRNA-regulated DNA walker for fluorescence detection of atherosclerosis-associated miRNAs. Reproduced from Ref [98]. Copyright 2026, Elsevier. (**b**) Schematic illustration of a particle-interacting three-dimensional DNA walker based on DNAzyme-functionalized walking particles for amplified fluorescence detection of HIV DNA. Reproduced from Ref [99]. Copyright 2026, Elsevier

More recently, DNA walkers have evolved into increasingly sophisticated dynamic nucleic acid circuits through integration with DNAzymes, CHA, and CRISPR-based molecular regulation systems. A CHA–RNA-cleaving DNAzyme junction-mediated circular-crRNA-regulated DNA walker was developed for the detection of atherosclerosis-associated miRNAs [98]. In this system, target miRNA first initiated CHA to assemble an active DNAzyme junction, which subsequently interacted with a circular crRNA regulatory module to activate DNA walker motion. The activated walker repeatedly performed cleavage and strand displacement reactions along the DNA track, allowing a single target molecule to trigger the activation of multiple fluorescent reporters. This programmable dynamic DNA circuit enabled highly sensitive and reliable quantification of atherosclerosis-related miRNAs, demonstrating that DNA walkers can function as complex fluorescence signal amplification networks rather than simple molecular motors (Fig. 6a). In addition, dynamic DNA amplification strategies have been further expanded through particle-interacting three-dimensional DNA walkers. A fluorescence biosensor designed for HIV DNA detection employed DNAzyme-functionalized walking particles together with fluorescent substrate particles [99]. Upon target recognition, the DNAzyme was activated to initiate walker motion, and the activated walker repeatedly cleaved fluorescent reporters immobilized on the substrate particles, resulting in fluorescence recovery. By converting a single target recognition event into multiple fluorescence generation cycles, this particle-interacting DNA walker achieved substantially higher signal amplification efficiency than conventional walker systems and enabled highly sensitive quantitative detection of HIV DNA (Fig. 6b).

In summary, dynamic DNA nanostructures have evolved into active fluorescence biosensing platforms capable of transforming a single molecular recognition event into repeated fluorescence activation through programmable strand displacement reactions and molecular motion. Early DNA walker systems have progressively developed into sophisticated amplification networks through integration with CHA, DNAzymes, metal nanoparticles, and CRISPR-based molecular circuits. Importantly, validation in human serum, blood, food-related samples, and bacterial genomic DNA further demonstrates the applicability of these dynamic DNA architectures to biologically and practically relevant sensing environments. Owing to their exceptional amplification efficiency and quantitative analytical performance, dynamic DNA nanostructures are expected to serve as a key enabling technology for next-generation ultrasensitive fluorescence biosensors. The analytical characteristics of representative fluorescent biosensing platforms incorporating dynamic DNA nanostructures, including target analytes, detection mechanisms, signal amplification strategies, LODs, assay times, and validation levels, are summarized in Table 6. 

**Table 6:** Comparison of dynamic DNA nanostructure-based fluorescence biosensing platforms

Architecture	Target	Mechanism	Amplification used	LOD	Assay time	Validation level	Ref.
Binding-induced 3D-bipedal DNA walker with CHA	Thrombin	Thrombin binding to two aptamer-containing feet, proximity-induced DNAzyme cleavage of track DNA, trigger DNA release, CHA, and fluorescence generation	3D-bipedal DNA walking and CHA	23 fM	> 90 min	10-fold diluted human serum	[93]
EXPAR-triggered 3D bipedal DNA walker on polystyrene microsphere tracks	miR-21	miR-21-triggered exponential amplification reaction (EXPAR), generation of BDW strands, assembly onto microsphere DNA tracks, nicking endonuclease-powered 3D bipedal walking, and fluorescence signal generation	EXPAR and 3D-bipedal DNA walking	5.2 fM	~ 70 min	10-fold diluted human serum	[94]
AuNP-based 3D DNA walker with enzyme-mediated CHA	*S. typhimurium*, *E. coli*	Bacterial target recognition, strand displacement, walker-strand release, 3D DNA walking on AuNP tracks, enzyme fragment generation, CHA, and fluorescence generation	3D DNA walking and CHA	22.9 CFU /mL (*S. typhimurium*), 28.1 CFU /mL (*E. coli*)	≤ 50 min	Chicken and milk	[95]
AuNP-based DNAzyme-powered 3D DNA walker + DNA tetrahedron-based CHA	Zika virus RNA sequence	ZIKV RNA recognition, DNAzyme-powered 3D DNA walking, track cleavage, trigger generation, localized CHA, and fluorescence recovery	3D DNA walking and localized CHA	20 pM	≤ 2 h	Diluted human blood sample	[92]
AuNP-conjugated single-legged DNA walker driven by CHA	*Klebsiella pneumoniae* target gene	Target DNA hybridization, strand displacement, CHA-mediated DNA walking on AuNPs, and AuNP spacing-dependent colorimetric change	CHA and single-legged DNA walking	2.5 nM	< 90 min	Bacterial genomic DNA	[96]
Target-responsive DNA TWJ with Nb.BbvCI-assisted DNA walking and MoS_2_ QD fluorescence reporter	DON	DON–aptamer binding, disruption of TWJ assembly, reduced Nb.BbvCI-assisted DNA walking, and altered MoS_2_ QD fluorescence	Nb.BbvCI-assisted DNA walking	9 pg/mL	~ 150 min	Cornmeal	[97]

## DNA origami-based fluorescence biosensors

DNA origami provides substantially greater structural complexity and spatial control than conventional DNA nanostructures, enabling the independent positioning of a wide variety of functional molecules at hundreds of addressable sites within a single nanostructure [9, 11]. Whereas conventional DNA nanostructures have primarily been utilized for structural assembly or localized signal amplification, DNA origami is distinguished by its ability to organize functional components with nanometer-scale precision, thereby enabling the rational design of optical signal generation and transduction [100]. This exceptional spatial programmability allows active control over fluorescence generation and signal amplification mechanisms, making DNA origami not merely a signal amplification platform but a versatile foundation for implementing diverse fluorescence biosensing strategies [52, 101].

DNA origami-based fluorescence biosensors can be broadly classified according to their signal transduction mechanisms into PEF [102], FRET-based sensing [101], and non-FRET fluorescence sensing [103]. Although all three strategies exploit the precise spatial organization provided by DNA origami, they differ fundamentally in the mechanisms by which fluorescence signals are generated and amplified. In this section, the design principles, signal transduction mechanisms, representative studies, and recent advances of each sensing strategy are discussed to provide a comprehensive overview of the development of DNA origami-based fluorescence biosensors.

### DNA origami nanoantenna-based pef biosensors

DNA origami-based plasmonic nanoantennas have emerged as powerful PEF platforms by precisely positioning fluorophores within the localized electromagnetic fields generated between plasmonic nanostructures [52]. Unlike conventionally fabricated plasmonic architectures with randomly distributed hotspots, DNA origami enables nanometer-scale control over the positions of metallic nanoparticles and the distance between nanoparticles and fluorophores, thereby creating highly uniform plasmonic coupling environments with excellent fluorescence enhancement efficiency and reproducibility [102]. This exceptional spatial addressability has facilitated the development of next-generation fluorescence biosensing platforms for the ultrasensitive detection of DNA, RNA, proteins, and small-molecule biomarkers.

**Fig. 7:**
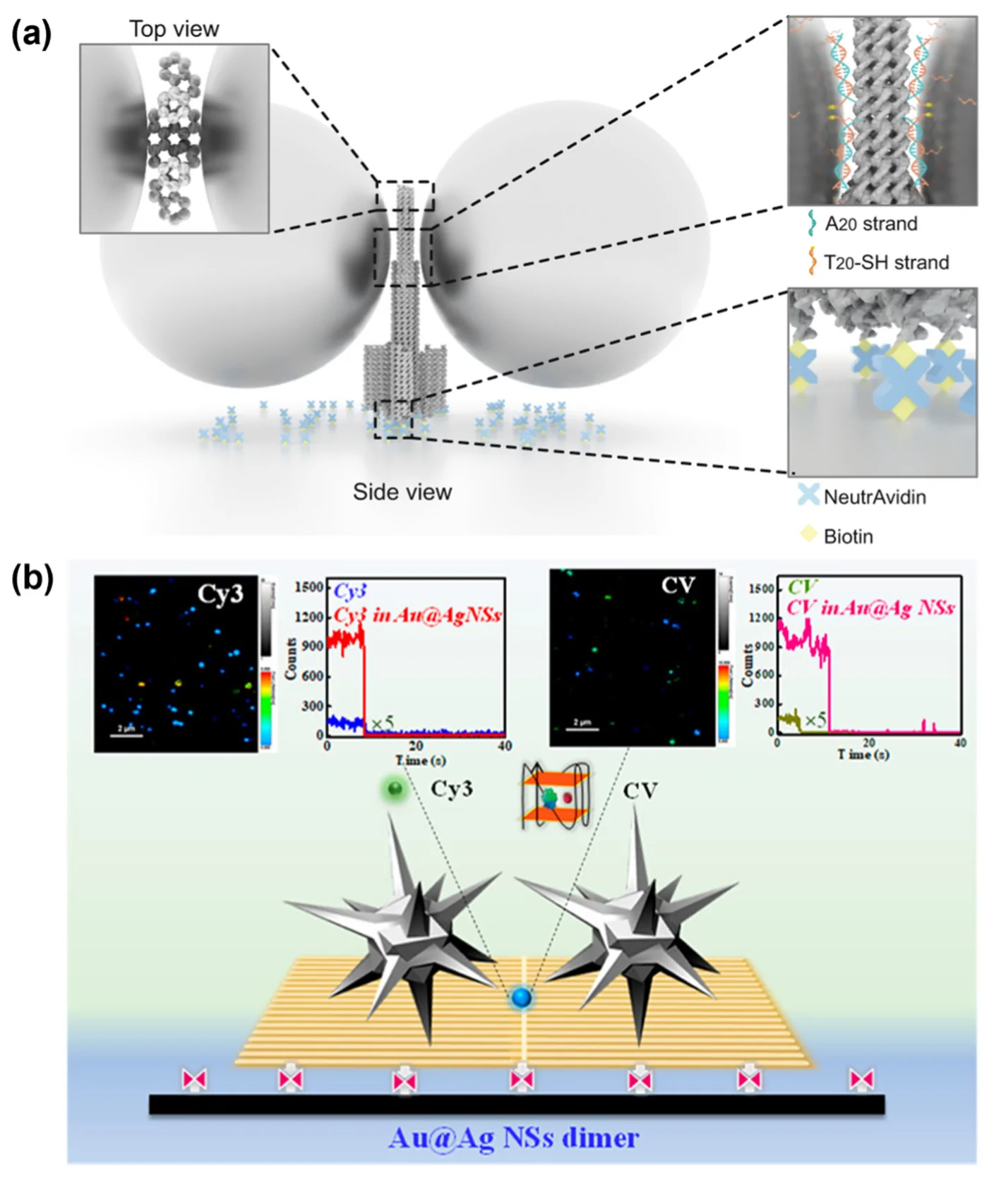
Representative applications of DNA origami nanoantennas-based PEF biosensing. (**a**) DNA origami-based NACHOS nanoantenna with cleared plasmonic hotspots for single-molecule fluorescence detection. Reproduced from Ref [104]. Licensed under CC BY 4.0. (**b**) DNA origami-assembled Au@Ag nanostar dimer nanoantenna for ATP detection through PEF. Reprinted with permission from [108]. Copyright 2023, American Chemical Society

A representative example is the NanoAntennas with Cleared HOtSpots (NACHOS) platform, which employed DNA origami to construct an open plasmonic nanoantenna architecture that allows both fluorophores and target analytes to directly access the plasmonic hotspot [104]. This open configuration maximized plasmon–fluorophore interactions, achieving fluorescence enhancement of up to 461-fold and enabling smartphone-based single-molecule fluorescence detection in a portable biosensing platform (Fig. 7a). The platform was further evaluated using human blood serum, demonstrating its potential for fluorescence-based detection in a biologically relevant sample matrix. This open nanoantenna concept was subsequently extended to the Trident nanoantenna design, which maintained strong fluorescence enhancement while further improving accessibility to the plasmonic hotspot and enhancing biomolecular binding efficiency [52].

DNA origami-based PEF nanoantennas have been broadly applied to the detection of nucleic acids, proteins, and small-molecule biomarkers. In one study employing nanopatterned DNA origami, sandwich hybridization was used to selectively position target DNA molecules within the plasmonic hotspot, enabling aM-level digital single-molecule detection through amplified fluorescence signals [105]. The nanopatterned nanoantenna platform was further validated using human blood plasma, with a detection limit of 10 aM, demonstrating its applicability to highly sensitive nucleic acid detection in a complex biological matrix. This work demonstrated that DNA origami nanoantennas can serve not only as fluorescence enhancement platforms but also as ultrasensitive nucleic acid sensing systems by precisely controlling molecular positioning. In another study, DNA origami-based plasmonic nanoantennas were employed for single-antibody detection, achieving approximately 17-fold fluorescence enhancement and demonstrating their potential for ultrasensitive immunoassays and multiplex biomarker analysis [102]. Furthermore, Au@Ag nanostar dimers were precisely assembled on DNA origami and integrated with an ATP recognition system to achieve single-molecule ATP detection [106]. The plasmonic hotspot generated fluorescence enhancement of up to 65-fold, highlighting the effectiveness of DNA origami nanoantennas for small-molecule biosensing applications (Fig. 7b).

More recently, DNA origami-based PEF platforms have expanded beyond conventional gold (Au)- and silver (Ag)-based plasmonic materials to incorporate alternative plasmonic nanostructures such as rhodium (Rh) [107]. DNA origami-directed Rh nanocube dimers generated plasmonic hotspots capable of efficiently enhancing the intrinsic deep-ultraviolet fluorescence of tryptophan residues in proteins. By positioning streptavidin molecules within these hotspots, label-free single-molecule detection based solely on protein autofluorescence was achieved. This study represents a significant extension of DNA origami-enabled PEF technology from fluorescence label-dependent biosensing toward intrinsic autofluorescence-based single-protein analysis by combining deep-UV plasmonics with the precise spatial programmability of DNA origami.

In summary, DNA origami-based plasmon-enhanced fluorescence platforms combine the precise spatial addressability of DNA origami with the extraordinary fluorescence enhancement capabilities of plasmonic nanostructures to achieve highly sensitive and reproducible fluorescence biosensing. These plasmonic nanoantennas have evolved from nucleic acid detection platforms to versatile biosensors capable of analyzing proteins, small molecules, and even single-molecule biomarkers, making them one of the most promising technologies for next-generation ultrasensitive fluorescence biosensing. The analytical characteristics of representative PEF-based biosensing platforms incorporating DNA origami nanoantennas, including target analytes, detection mechanisms, signal amplification strategies, LODs, assay times, and validation levels, are summarized in Table 7. 

**Table 7:** Comparison of DNA origami nanoantenna-based PEF biosensing platforms

Architecture	Target	Mechanism	LOD	Assay time	Validation level	Ref.
Tower DNA origami nanoantenna with two AgNPs	34 nt OXA-48 gene-specific DNA	Sandwich hybridization, AF647 localization in the plasmonic hotspot, and PEF	-	> 2 h	Human blood serum	[104]
Trident DNA origami nanoantenna with two AgNPs	151 nt OXA-48 gene-specific DNA	Sandwich hybridization, AF647 localization in the plasmonic hotspot, and PEF	-	10–40 min	-	[52]
Nanopatterned trident DNA origami nanoantennas with two AgNPs, site-specifically placed via trangle DNA origami	151 nt OXA-48 gene-specific DNA	Sandwich hybridization with microfluidic-assisted target capture, AF647 localization in the plasmonic hotspot, and PEF	5 aM (in buffer), 10 aM (in human blood plasma)	45–60 min	Human blood plasma	[105]
DNA origami nanoantenna with an antibody-responsive DNA switch	Anti-digoxigenin antibody	Antibody-induced fluorescence activation	-	~ 20 min	-	[102]
DNA origami-assembled Au@Ag nanostar dimer nanoantenna with an ATP-responsive aptamer	ATP	ATP-induced aptamer folding and fluorescence enhancement	-	~ 6 h	-	[106]
DNA origami-assembled Rh nanocube dimer nanoantenna	Streptavidin	Deep-UV plasmon-enhanced protein autofluorescence	-	~ 157 min	-	[107]

### FRET-based DNA origami fluorescence biosensors

The exceptional structural programmability of DNA origami has made it an ideal platform for FRET-based fluorescence biosensing, where precise spatial organization of optical components is essential. FRET is a distance-dependent energy transfer process in which the efficiency of non-radiative energy transfer between a donor and an acceptor changes sensitively with their separation distance [37]. In DNA origami architectures, subtle structural rearrangements induced by target binding can therefore be converted into quantitative fluorescence signals [108]. Unlike freely diffusing DNA probes, DNA origami enables nanometer-scale control over the relative positions and orientations of fluorophore–acceptor or fluorophore–quencher pairs, thereby minimizing nonspecific background signals and achieving a high signal-to-background ratio [18, 109]. Owing to these advantages, DNA origami-based FRET sensors have evolved into highly sensitive fluorescence biosensing platforms by integrating structural reconfiguration, strand displacement, aptamer-mediated recognition, and other programmable molecular recognition mechanisms for the quantitative detection of nucleic acids and protein biomarkers.

Early studies primarily focused on converting DNA origami structural reconfiguration into FRET signals. A representative example is an ATP-responsive DNA origami lever that incorporated a split aptamer to translate ATP-induced conformational changes into changes in the donor–acceptor distance, thereby enabling quantitative ATP detection through FRET [110]. Similarly, an aptamer-functionalized DNA origami nanobox exploited target-induced opening of a closed nanostructure upon binding of a specific biomolecule [111]. The corresponding FRET signal enabled the nanobox to function as a molecular container for fluorescence detection of *Plasmodium falciparum* lactate dehydrogenase (PfLDH).

**Fig. 8:**
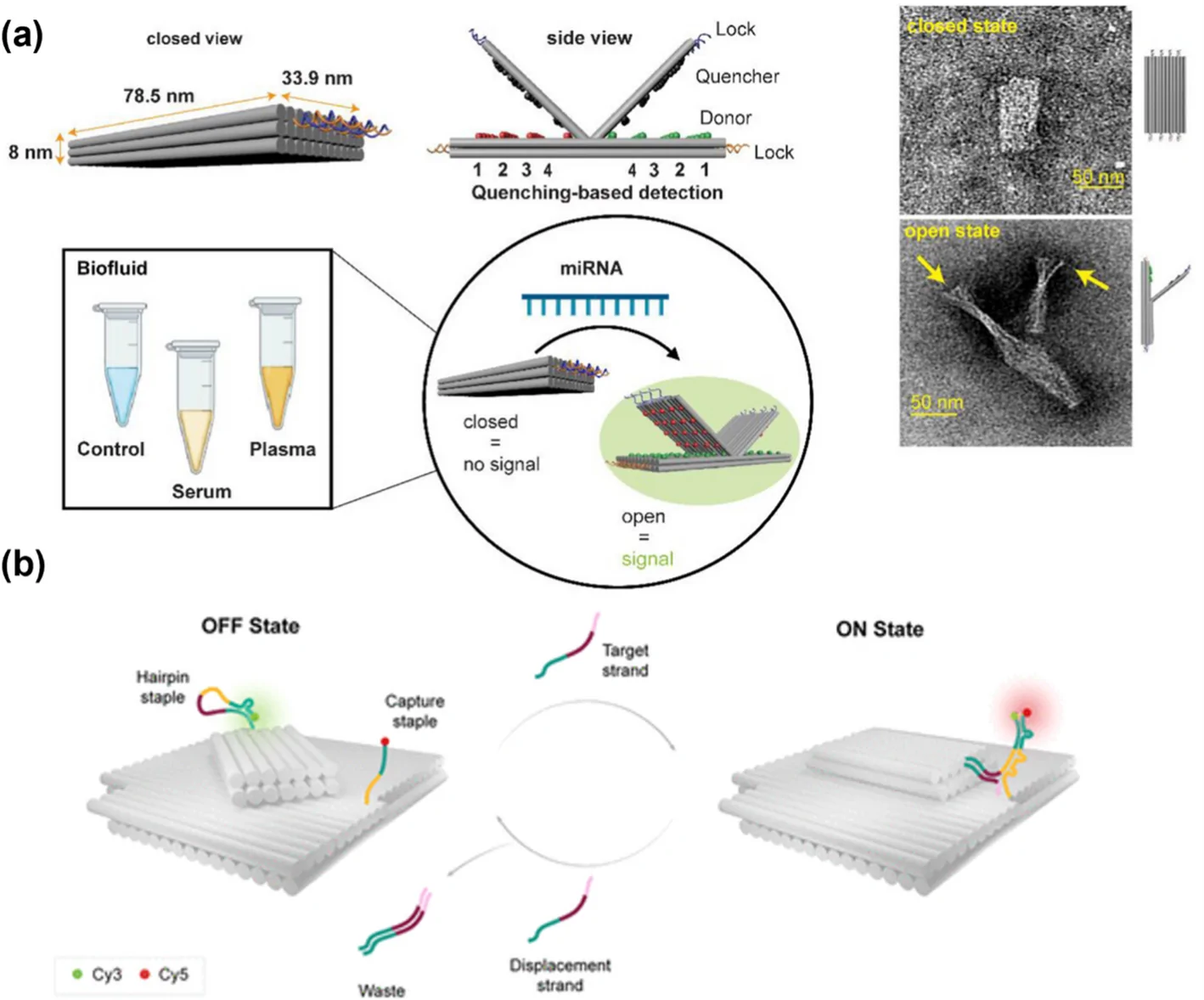
Representative applications of DNA origami-based FRET biosensing. (**a**) DNA origami book biosensor enabling target-responsive conformational switching and fluorescence modulation for miRNA sensing. Reproduced from Ref [101]. Licensed under CC BY 4.0. (**b**) Reconfigurable rotating DNA origami nanodevice for reversible FRET and fluorescence quenching-based sensing. Reprinted with permission from [114]. Copyright 2026, American Chemical Society

Subsequently, DNA origami-based FRET biosensors evolved toward platforms that combine structural opening/closing with strand displacement reactions. In a DNA origami hinge structure, target nucleic acids initiated strand displacement, triggering structural reconfiguration and restoring fluorescence that had previously been quenched, thereby enabling sensitive nucleic acid detection [44]. A DNA origami “book” architecture further exploited reversible opening and closing motions to simultaneously arrange multiple fluorophore–quencher pairs, enabling fluorescence modulation and multiplexed target analysis upon target recognition [109]. The sensing capability of this DNA origami book was further demonstrated using RNA extracted from MCF-7 cells, supporting its applicability to the analysis of biologically derived nucleic acids. More recently, the DNA origami book sensor was further optimized by incorporating locked nucleic acid (LNA) and 2′-fluoro-modified recognition sequences together with stabilized linkers [101]. This improved platform enabled direct detection of circulating miRNAs in serum and plasma without enzymatic amplification, extending DNA origami-based FRET sensing to liquid biopsy applications (Fig. 8a).

DNA origami-based FRET biosensors have also been applied to single-molecule biological analysis. By precisely controlling the position and orientation of individual aptamers, DNA origami has enabled highly reliable single-molecule FRET (smFRET) fluorescence biosensing. More recently, DNA origami-based FRET sensors have evolved into dynamic nanodevices with reusable sensing capabilities. A reconfigurable rotating DNA origami nanodevice utilized target-induced TMSD to generate controlled rotational motion, which altered the distances between fluorophore–acceptor or fluorophore–quencher pairs and produced measurable FRET or fluorescence quenching signals [112]. Furthermore, reversible strand exchange enabled repeated target detection, overcoming the single-use limitation of conventional DNA origami sensors and demonstrating a regenerable fluorescence sensing platform (Fig. 8b). Importantly, the optimized sensor was validated using human serum and plasma, including clinical plasma samples, demonstrating its potential for direct miRNA analysis in liquid biopsy-related samples.

Beyond molecular detection, DNA origami-based FRET platforms have also been employed for single-molecule analysis of the physicochemical properties of biological membranes. DNA origami enables nanometer-scale positioning of fluorophores and membrane-binding modules, allowing subtle membrane-induced structural changes to be quantitatively converted into FRET signals. A representative example is a DNA origami-based single-molecule voltage sensor, in which an acceptor embedded within a lipid membrane and a donor attached to the DNA origami were precisely positioned to monitor membrane potential through changes in donor–acceptor distance [113]. This design enabled non-genetically encoded single-molecule membrane potential measurements. The same concept was subsequently extended to membrane surface charge sensing. A rectangular DNA origami carrying cholesterol anchors and a FRET-based charge-sensing module converted lipid membrane surface potential changes into structural rearrangements that could be quantified by FRET, allowing single-molecule analysis of membrane surface charge across lipid bilayers of different compositions [114]. More recently, DNA origami membrane sensors have been expanded into multifunctional nanodevices. One study reported a DNA origami platform attached to lipid vesicles that employed smFRET to monitor membrane binding and subsequently delivered sensing strands through TMSD, demonstrating the potential of DNA origami to integrate biosensing and molecular cargo delivery within a single programmable platform [115]. In addition, a DNA origami curvature sensor was developed to convert DNA origami deformation into FRET signals, enabling quantitative single-molecule measurements of the curvature of nanoparticles and lipid vesicles ranging from 50 to 300 nm in diameter [116]. These membrane and nanostructure sensing platforms were demonstrated using model systems including large unilamellar vesicles (LUVs), lipid bilayers, silica nanoparticles, and lipid vesicles, highlighting the versatility of DNA origami-based FRET sensors for quantitative analysis of membrane properties and nanoscale structural features.

In summary, DNA origami-based FRET biosensors have evolved into versatile platforms that exploit the nanometer-scale spatial addressability of DNA origami to precisely control donor–acceptor and fluorophore–quencher distances, thereby converting molecular recognition events and structural reconfiguration into quantitative fluorescence signals. Beginning with simple conformational switching sensors, these platforms have progressively expanded to incorporate strand displacement, aptamer-mediated recognition, single-molecule FRET analysis, and investigations of the electrical and physical properties of biological membranes. Consequently, DNA origami-based FRET sensors are now capable of monitoring not only biomolecular targets but also the dynamic behavior of biological membranes and nanostructures in real time. Furthermore, the exceptional structural programmability of DNA origami enables precise spatial organization of fluorescent components, providing high signal-to-background ratios and excellent quantitative performance without requiring complex signal amplification strategies. Validation in biological and model systems further demonstrates the broad applicability of DNA origami-based FRET platforms. These unique advantages position DNA origami-based FRET platforms as next-generation programmable fluorescence biosensors capable of both ultrasensitive biomolecular detection and single-molecule biophysical analysis.The analytical characteristics of representative FRET-based DNA origami biosensing platforms, including target analytes, detection mechanisms, signal amplification strategies, LODs, assay times, and validation levels, are summarized in Table 8. 

**Table 8:** Comparison of FRET-based DNA origami fluorescence biosensing platforms

Architecture	Target	Mechanism	Amplification used	LOD	Assay time	Validation level	Ref.
DNA origami with a split ATP aptamer	ATP	ATP-induced DNA origami closure, FRET-based green-to-red fluorescence change	-	-	-	-	[110]
DNA origami nanobox with a PfLDH aptamer-based lock	PfLDH	PfLDH–aptamer binding, aptamer conformational change and strand displacement, nanobox opening, increased Cy3–Cy5 distance, and decreased FRET	-	-	~ 180 min	-	[111]
Hinge-like DNA origami beacon with multifluorophore donor/acceptor arrays	Target ssDNA	Target DNA-mediated strand displacement, beacon opening, increased donor–acceptor distance, and decreased FRET	Multifluorophore FRET	-	< 45 min	-	[44]
Dynamic DNA origami book biosensor with FRET pairs	ODN-153, ODN-342, miR-21, let-7a	Target-induced strand displacement, DNA origami upper layer opening and rotation, and fluorescence change	Multifluorophore FRET	1–10 pM	< 10 min	MCF-7 cell-derived RNA	[109]
Dynamic DNA origami book with fluorophore–quencher arrays and lock strands	miR-21, miR-7a	Target-induced strand displacement, DNA origami book opening, and FRET decrease	Multifluorophore FRET	0.69 pM (miR-21 in buffer), 8.86 pM (miR-21 in 100% serum), 23.24 pM (miR-21 in 100% plasma)	< 10 min	30/50/100% human serum, 30/50/100% human plasma and clinical plasma	[101]
Reconfigurable rotating DNA origami nanodevice	Nucleic acid	Target-induced strand displacement, top-bundle rotation and locking, and FRET	-	0.71 nM	~ 1 h	-	[112]
Rectangular DNA origami-based membrane voltage sensor	Transmembrane potential (ΔΨ)	Voltage-induced displacement and conformational change of the anionic donor, change in donor–acceptor distance, and FRET change	-	-	~ 130 min	LUVs	[113]
DNA origami plate-based membrane surface-charge sensor	Membrane surface charge and surface potential	Surface charge-dependent conformational change of the anionic ssDNA sensing unit and FRET change	-	-	Overnight	LUVs	[114]
Rectangular DNA origami vesicle sensor	LUVs (vesicle binding)	Vesicle binding, ssDNA leash extension toward the lipid bilayer, increased donor–acceptor distance, and decreased FRET	-	-	~ 1 h	LUVs	[115]
Segmented DNA origami curvature sensor with flexible DNA linkers	Nanoparticle and lipid vesicle surface curvature		-	-	-	Silica nanoparticles and lipid vesicles	[116]

### Non-FRET DNA origami fluorescence biosensors

Unlike FRET-based sensors, DNA origami can function as a structural scaffold for the precise arrangement of fluorophores, removal of quenchers, activation of fluorescent reporters, and organization of enzyme-mediated signal amplification reactions, thereby enabling a wide range of fluorescence biosensing platforms without relying on FRET [117]. Owing to its exceptional spatial addressability, DNA origami allows multiple fluorescent probes and target-recognition elements to be positioned with nanometer precision within a single nanostructure.

**Fig. 9:**
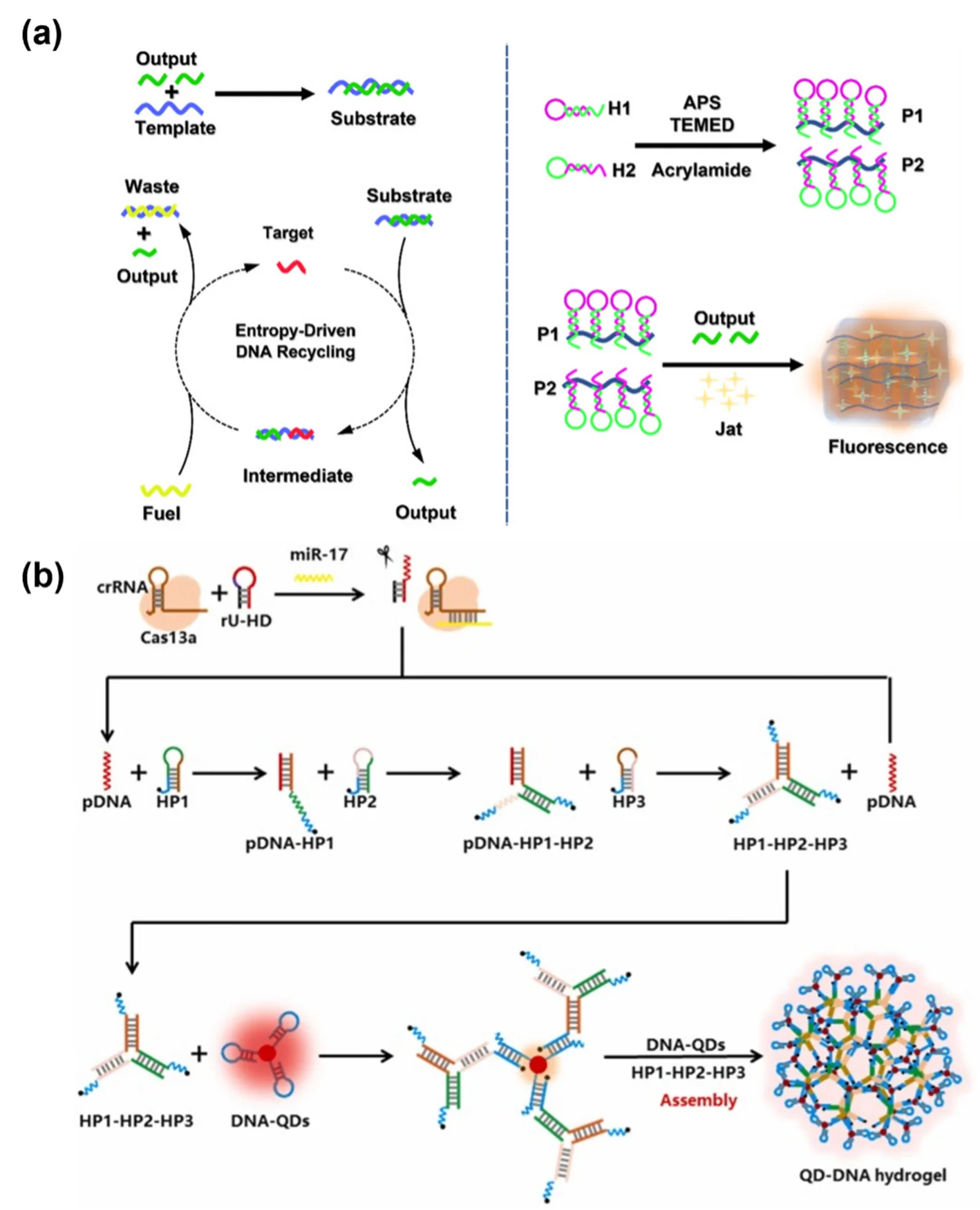
Representative applications of DNA origami-based non-FRET biosensing. (**a**) DNA origami-based fluorescent reporter platform organizing multiple fluorophore-labeled probes for amplified target detection. Reproduced from Ref [103]. Licensed under CC BY 4.0. (**b**) DNA origami-based localized CHA platform for amplified fluorescence detection of intracellular miR-21. Reproduced from Ref [118]. Copyright 2021, Elsevier

One representative application utilizes DNA origami as a scaffold for organizing multiple fluorescent reporters [103]. In a DNA origami-based reporter platform, multiple fluorophore-labeled DNA probes and target-recognition sequences were integrated into a single nanostructure, generating substantially stronger fluorescence signals than conventional single-stranded DNA (ssDNA) probes and achieving detection sensitivity at the fM level (Fig. 9a). In another study, a three-dimensional DNA origami scaffold was employed to spatially organize aptamers, thereby optimizing their orientation and accessibility [14]. This arrangement significantly improved the binding efficiency and signal-to-background ratio of sandwich fluorescence immunoassays.

DNA origami has also been employed as a confined reaction platform for fluorescence signal amplification. In a fluorescence biosensor incorporating CHA circuits localized on the DNA origami surface, the spatial confinement of reaction components shortened the distance between hairpin probes, thereby accelerating reaction kinetics and improving amplification efficiency [118]. In this system, the cancer-associated miRNA miR-21 triggered hairpin activation, resulting in the removal of quenchers and subsequent fluorescence recovery, enabling highly sensitive intracellular miRNA detection. The sensing performance was demonstrated in live cells, highlighting the potential of DNA origami for intracellular fluorescence biosensing. In addition, DNA origami served as a stable carrier for nucleic acid probes, protecting them from nuclease degradation and enhancing the overall stability of the sensing platform (Fig. 9b).

DNA origami has also been utilized as a spatially programmable scaffold for quenching-based fluorescence biosensing, in which fluorophore–quencher interactions are precisely organized to regulate fluorescence generation. In a duplexed cortisol aptasensor, aptamer–quencher assemblies were spatially organized on the DNA origami surface to stabilize the sensing configuration [119]. Upon cortisol binding, aptamer reconfiguration displaced the quencher and generated fluorescence recovery. This nanoscale spatial organization effectively reduced aptamer leakage and enabled real-time single-molecule biomarker detection. Building on this concept, DNA origami has further been utilized as a fluorescence sensing interface for monitoring molecular interactions occurring on the cell surface in real time [117]. In the cell sensing platform, strand displacement-based fluorophore–quencher sensors were spatially organized on DNA origami structures anchored to the plasma membrane. Upon recognition of the target nucleic acid, the quencher-labeled strand was displaced, resulting in fluorescence recovery and enabling real-time monitoring of extracellular nucleic acid dynamics in the vicinity of living cells. The platform was demonstrated in living cells and extracellular matrix environments, highlighting its applicability to fluorescence monitoring in biologically relevant settings.

A further direction in DNA origami-based fluorescence biosensing involves engineering dynamic nanostructures that convert target-induced conformational changes directly into fluorescence signals. A representative example is the bioinspired DNA NanoGripper, a flexible DNA origami nanodevice designed to capture viral particles [120]. Binding between viral spike proteins and aptamers induced conformational changes in the NanoGripper, which were subsequently translated into fluorescence signals. By integrating a photonic crystal-based fluorescence enhancement strategy, the generated fluorescence was further amplified, enabling highly sensitive detection of SARS-CoV-2. The platform was further validated using human saliva, demonstrating its applicability to direct viral detection in a clinically relevant sample matrix. This study demonstrated that DNA origami can integrate target-responsive structural reconfiguration with optical signal enhancement to create highly sensitive fluorescence biosensing platforms.

In summary, DNA origami-based fluorescence biosensors exploit the nanometer-scale structural programmability of DNA origami to precisely organize fluorophores, regulate fluorescence generation and signal transduction, and localize target-responsive reaction components, thereby achieving enhanced fluorescence intensity and high analytical sensitivity. Early studies primarily focused on signal enhancement through the spatial organization of fluorescent reporters, whereas more recent developments have incorporated confined nucleic acid amplification circuits and structure-responsive fluorescence activation within DNA origami architectures. Furthermore, DNA origami has evolved into a versatile fluorescence imaging platform capable of monitoring molecular interactions on cell surfaces and within complex biological environments in real time. Demonstration in biological and complex sample systems further highlights the practical applicability of DNA origami-based fluorescence biosensors. Collectively, these advances demonstrate that DNA origami functions not merely as a passive scaffold for fluorophore arrangement, but as a programmable optical nanoplatform capable of actively regulating fluorescence generation, amplification, and modulation. Combined with emerging signal amplification strategies, DNA origami is expected to play a pivotal role in the development of next-generation fluorescence biosensors for ultrasensitive and real-time bioanalysis. The analytical characteristics of representative non-FRET DNA origami biosensing platforms, including target analytes, detection mechanisms, signal amplification strategies, LODs, assay times, and validation levels, are summarized in Table 9. 

**Table 9:** Comparison of non-FRET DNA origami fluorescence biosensing platforms

Architecture	Target	Mechanism	Amplification used	LOD	Assay time	Validation level	Ref.
Triangular DNA origami reporter with dye molecules and multiple target-binding probes	Oligonucleotide	Capture probe–target hybridization, DNA origami reporter recruitment, and fluorescence enhancement	Multifluorophore	78 pM (single target) 2.7–9.5 pM (multiplex)	> overnight	-	[103]
DNA origami 24-helix bundle nanopatterning an encoded microparticle surface with spatially positioned aptamers	Thrombin	DNA-origami-controlled aptamer spacing and orientation, reduced steric hindrance and increased accessibility, enhanced thrombin binding, and fluorescent sandwich detection	-	2 nM	~ 40 min	10-fold diluted plasma	[14]
Rectangular DNA origami with spatially organized CHA hairpins	miR-21	miR-21-triggered hairpin opening, catalytic hairpin assembly, repeated amplification cycles, and fluorescence recovery	CHA	20.9 pM	~ 20 min	Live cells	[118]
Triangular DNA origami with a single cortisol-responsive duplexed aptasensor immobilized on a glass surface	Cortisol	Cortisol binding, aptamer conformational change and dehybridization, quencher release, and fluorescence recovery	-	-	~ 7.5 min	-	[119]
DNA origami cell sensing platform with spatially organized ssDNA overhangs and fluorescent reporters	ssDNA targets	Target DNA hybridization to complementary probes on CSP, quencher strand displacement, fluorescence activation, and simultaneous detection of two targets through spatially separated probes	-	-	~ 15 min	Live cells, extracellular matrix	[117]
DNA origami NanoGripper with four bendable fingers and multiple aptamer nanoswitches	Intact SARS-CoV-2 virions	Spike protein–targeting aptamer binding, BHQ-labeled quencher displacement, fluorescence activation, photonic crystal-enhanced fluorescence, and digital counting	photonic crystal fluorescence enhancement	100 genome copies/mL	~ 30 min	Human saliva	[120]

## Challenges and future perspectives

DNA nanostructure- and DNA origami-based fluorescence biosensors have advanced rapidly owing to their high sensitivity, structural programmability, and versatile functional capabilities. However, several technical challenges related to structural stability, manufacturing scalability, signal reproducibility, and clinical applicability must be addressed before these platforms can be translated into practical diagnostic technologies.

One of the major challenges is maintaining the structural stability of DNA nanostructures under physiological conditions. DNA nanostructures are susceptible to nuclease degradation and structural instability caused by physiological ionic conditions and pH fluctuations. These issues are particularly critical for dynamic DNA nanostructures that rely on target-induced conformational changes for signal generation. Although protective strategies such as silica coating [105], and polymer-based coating [121, 122] have been developed to improve structural stability, these approaches may reduce the accessibility of the DNA nanostructure surface and limit its functional addressability. Therefore, new protection strategies that preserve the precise spatial programmability of DNA origami while enhancing biostability are required. In addition, manufacturing-related challenges, including the high cost of chemically modified oligonucleotides, limited DNA origami folding yield, and the lack of standardized large-scale production processes, must also be overcome to facilitate practical diagnostic applications.

Ensuring signal reproducibility and quantitative analytical reliability remains another significant challenge. Minor variations in the self-assembly process can result in batch-to-batch variability in DNA nanostructure-based sensors. This issue is particularly pronounced in PEF platforms, where fluorescence enhancement is highly sensitive to the size, morphology, and interparticle spacing of metallic nanostructures [123]. Similarly, in FRET-based sensors, variations in fluorophore labeling efficiency and DNA nanostructure assembly can increase background signals and compromise quantitative accuracy [124]. To address these limitations, standardized characterization methods, rigorous quality control criteria, and reliable quantitative calibration systems should be established. Furthermore, to overcome the intrinsic photophysical limitations of conventional fluorophores, including photobleaching and fluorescence blinking, integrating DNA nanostructures with highly photostable optical materials such as QDs, upconversion nanoparticles (UCNPs), and metal nanoclusters represents a promising strategy.

Maintaining analytical performance in complex biological samples and achieving clinical applicability also remain critical challenges. Although DNA nanostructure-based fluorescence biosensors exhibit excellent analytical sensitivity under controlled laboratory conditions, their performance may deteriorate in complex biological matrices such as blood, serum, and saliva because of nonspecific interactions and matrix effects, which reduce signal accuracy and reproducibility [125, 126]. Consequently, successful clinical translation will require improved target selectivity, simplified sample preparation procedures, integration with portable fluorescence readout systems, and extensive validation using diverse clinical specimens.

Looking forward, DNA nanostructure-based fluorescence biosensors are expected to evolve further through integration with emerging materials and advanced data analysis technologies. Machine learning- and artificial intelligence-based fluorescence image analysis can improve signal interpretation accuracy and enhance multiplex detection capabilities [127, 128]. In addition, combining DNA nanostructure-based sensors with microfluidic systems and lateral flow assays may enable fully integrated sample-to-answer diagnostic platforms suitable for point-of-care testing [129]. The incorporation of emerging nanomaterials—including two-dimensional materials such as MXenes, Rh-based nanoantennas operating in the deep-ultraviolet region, and photonic crystal-based fluorescence enhancement structures—with DNA origami is also expected to significantly improve sensitivity while expanding the accessible optical detection range.

Ultimately, the future development of DNA nanostructure-based fluorescence biosensors will depend on the convergence of precisely programmable nanostructure engineering, advanced optical nanomaterials, artificial intelligence-assisted data analysis, and platform designs optimized for real-world clinical applications. Such interdisciplinary advances are expected to transform DNA nanotechnology from a molecular detection platform into a next-generation biosensing technology capable of ultrasensitive detection, multiplexed analysis, and practical clinical diagnostics.

## Conclusions

DNA nanostructure- and DNA origami-based fluorescence biosensing platforms have rapidly advanced by exploiting the inherent programmability of DNA to precisely organize functional components at the nanoscale. In this review, we summarized recent progress in fluorescence biosensors based on diverse DNA nanostructures, including TWJs, hyperbranched and dendritic DNA architectures, DNA hydrogels, tetrahedral DNA nanostructures (TDNs), and dynamic DNA nanodevices. These platforms enhance fluorescence sensing performance by enabling programmable structural organization, target-responsive conformational regulation, and efficient signal amplification. In addition, DNA origami-based fluorescence biosensors were categorized according to their signal transduction mechanisms, including PEF, FRET-based sensing, and non-FRET fluorescence strategies, highlighting the unique capability of DNA origami to precisely control optical interactions at the nanoscale.

Recent advances have demonstrated that DNA nanotechnology has evolved beyond the role of a passive structural scaffold into an active and programmable sensing platform. Through precise spatial arrangement of fluorophores, quenchers, recognition elements, enzymes, and nanomaterials, DNA nanostructures enable improved fluorescence signal generation, enhanced signal-to-background ratios, and highly quantitative detection of diverse biomolecular targets. In particular, DNA origami provides unprecedented spatial addressability, allowing precise regulation of fluorophore–fluorophore, fluorophore–metal, and donor–acceptor interactions, thereby enabling sophisticated fluorescence modulation strategies that are difficult to achieve with conventional sensing platforms.

Despite significant progress, further efforts are required to overcome challenges associated with structural stability, scalable manufacturing, signal reproducibility, and translation into practical diagnostic applications. Future development will likely be driven by the integration of programmable DNA nanotechnology with advanced optical materials, computational design approaches, artificial intelligence-assisted analysis, and portable diagnostic platforms. Ultimately, DNA nanostructures and DNA origami are expected to serve as versatile molecular engineering platforms for controlling nanoscale interactions and enabling next-generation fluorescence biosensors with improved sensitivity, multiplexing capability, and clinical applicability.

## Data Availability

No datasets were generated or analysed during the current study.

## References

[1] Selleck MJ, Senthil M, Wall NR. Making Meaningful Clinical Use of Biomarkers. Biomark Insights. 2017;12:117727191771523. 10.1177/1177271917715236. PMC5479428 28659713

[2] Bhatia D, Paul S, Acharjee T, Ramachairy SS. Biosensors and Their Widespread Impact on Human Health. Sens Int. 2024;5:100257. 10.1016/j.sintl.2023.100257.

[3] Turner APF, Biosensors. Sense and Sensibility. Chem Soc Rev. 2013;42:3184. 10.1039/c3cs35528d. 23420144

[4] Babendure JR, Adams SR, Tsien RY. Aptamers Switch on Fluorescence of Triphenylmethane Dyes. J Am Chem Soc. 2003;125:14716–7. 10.1021/ja037994o. 14640641

[5] Gest AMM, Sahan AZ, Zhong Y, Lin W, Mehta S, Zhang J. Molecular Spies in Action: Genetically Encoded Fluorescent Biosensors Light up Cellular Signals. Chem Rev. 2024;124:12573–660. 10.1021/acs.chemrev.4c00293. PMC11613326 39535501

[6] Lu C-H, Willner B, Willner IDNA, Nanotechnology. From Sensing and DNA Machines to Drug-Delivery Systems. ACS Nano. 2013;7:8320–32. 10.1021/nn404613v. 24070191

[7] Wamhoff E-C, Banal JL, Bricker WP, Shepherd TR, Parsons MF, Veneziano R, Stone MB, Jun H, Wang X, Bathe M. Programming Structured DNA Assemblies to Probe Biophysical Processes. Annu Rev Biophys. 2019;48:395–419. 10.1146/annurev-biophys-052118-115259. PMC7035826 31084582

[8] He Z, Shi K, Li J, Chao J. Self-Assembly of DNA Origami for Nanofabrication, Biosensing, Drug Delivery, and Computational Storage. iScience. 2023;26:106638. 10.1016/j.isci.2023.106638. PMC10176269 37187699

[9] Rothemund PWK. Folding DNA to Create Nanoscale Shapes and Patterns. Nature. 2006;440:297–302. 10.1038/nature04586. 16541064

[10] Kogikoski S, Ameixa J, Mostafa A, Bald I. Lab-on-a-DNA Origami: Nanoengineered Single-Molecule Platforms. Chem Commun. 2023;59:4726–41. 10.1039/D3CC00718A. PMC10111202 37000514

[11] Saccà B, Niemeyer CMDNA, Origami. The Art of Folding DNA. Angew Chem Int Ed. 2012;51:58–66. 10.1002/anie.201105846. 22162047

[12] Meyer R, Niemeyer CM. Orthogonal Protein Decoration of DNA Nanostructures. Small. 2011;7:3211–8. 10.1002/smll.201101365. 21953812

[13] Lee JB, Roh YH, Um SH, Funabashi H, Cheng W, Cha JJ, Kiatwuthinon P, Muller DA, Luo D. Multifunctional Nanoarchitectures from DNA-Based ABC Monomers. Nat Nanotechnol. 2009;4:430–6. 10.1038/nnano.2009.93. PMC7097395 19581895

[14] Rutten I, Daems D, Lammertyn J. Boosting Biomolecular Interactions through DNA Origami Nano-Tailored Biosensing Interfaces. J Mater Chem B. 2020;8:3606–15. 10.1039/C9TB02439E. 31922167

[15] Domljanovic I, Carstens A, Okholm A, Kjems J, Nielsen CT, Heegaard NHH, Astakhova K. Complexes of DNA with Fluorescent Dyes Are Effective Reagents for Detection of Autoimmune Antibodies. Sci Rep. 2017;7:1925. 10.1038/s41598-017-02214-0. PMC5432514 28507286

[16] Daems D, Rutten I, Bath J, Decrop D, Van Gorp H, Ruiz EP, De Feyter S, Turberfield AJ, Lammertyn J. Controlling the Bioreceptor Spatial Distribution at the Nanoscale for Single Molecule Counting in Microwell Arrays. ACS Sens. 2019;4:2327–35. 10.1021/acssensors.9b00877. 31436077

[17] Choi Y, Kotthoff L, Olejko L, Resch-Genger U, Bald IDNA. Origami-Based Förster Resonance Energy-Transfer Nanoarrays and Their Application as Ratiometric Sensors. ACS Appl Mater Interfaces. 2018;10:23295–302. 10.1021/acsami.8b03585. 29916243

[18] Li Y, Du H, Wang W, Zhang P, Xu L, Wen Y, Zhang XA. Versatile Multiple Target Detection System Based on DNA Nano-Assembled Linear FRET Arrays. Sci Rep. 2016;6:26879. 10.1038/srep26879. PMC4882533 27230484

[19] Zhou X, Zhao M, Duan X, Guo B, Cheng W, Ding S, Ju H. Collapse of DNA Tetrahedron Nanostructure for Off–On Fluorescence Detection of DNA Methyltransferase Activity. ACS Appl Mater Interfaces. 2017;9:40087–93. 10.1021/acsami.7b13551. 29111659

[20] Ahmadi-Sangachin E, Hosseini M, Mohammadnejad JMRNA. Sensing Using Integrating DNA-Functionalized Hydrogels with Aggregation-Induced Emission of Silver Nanoclusters. Sens Actuators B Chem. 2023;390:134012. 10.1016/j.snb.2023.134012.

[21] Sheng J, Deng R, Feng L, Niu B, Gong M, Zhao S, Xie Z, Xie S, Tang Y, Luo J, et al. De Novo-Engineered Circular DNAzymes with Topological Rigidity for Multiplexed Fluorescent-Temporal Barcoding. Chem Eng J. 2026;538:176705. 10.1016/j.cej.2026.176705.

[22] Ochmann SE, Vietz C, Trofymchuk K, Acuna GP, Lalkens B, Tinnefeld P. Optical Nanoantenna for Single Molecule-Based Detection of Zika Virus Nucleic Acids without Molecular Multiplication. Anal Chem. 2017;89:13000–7. 10.1021/acs.analchem.7b04082. 29144729

[23] Puchkova A, Vietz C, Pibiri E, Wünsch B, Sanz Paz M, Acuna GP, Tinnefeld P. DNA Origami Nanoantennas with over 5000-Fold Fluorescence Enhancement and Single-Molecule Detection at 25 ΜM. Nano Lett. 2015;15:8354–9. 10.1021/acs.nanolett.5b04045. 26523768

[24] Liu B, Wang F, Chao J. Programmable Nanostructures Based on Framework-DNA for Applications in Biosensing. Sens 2023. 2023;23:3313. 10.3390/S23063313. PMC10051322 36992023

[25] Zahraee H, Khoshbin Z, Mohammadi F, Mashreghi M, Abnous K, Taghdisi SM. Three-Way Junction Skeleton Biosensors Based on Aptamers, DNAzymes, and DNA Hybridization Probes. TRAC Trends Anal Chem. 2023;165:117160. 10.1016/j.trac.2023.117160.

[26] Qi Z, Wei C, Zhang F, Wang Z, Zuo X. Tetrahedral DNA Frameworks for Biosensing and Imaging Analysis in Living Cells. Nano Today. 2024;54:102127. 10.1016/j.nantod.2023.102127.

[27] Qiu Y, Qiu Y, Zhou W, Lu D, Wang H, Li B, Liu B, Wang W. Advancements in Functional Tetrahedral DNA Nanostructures for Multi-Biomarker Biosensing: Applications in Disease Diagnosis, Food Safety, and Environmental Monitoring. Mater Today Bio. 2025;31:101486. 10.1016/j.mtbio.2025.101486. PMC11810847 39935897

[28] Zhang P, Ouyang Y, Zhuo Y, Chai Y, Yuan R. Recent Advances in DNA Nanostructures Applied in Sensing Interfaces and Cellular Imaging. Anal Chem. 2023;95:407–19. 10.1021/acs.analchem.2c04540. 36625113

[29] Sarwar A, Shakeel F, Fatima T, Amin R, Rizvi SNB, Hussain T, Afzal A. DNA Nanotechnology for Next-Generation Biosensors: Principles, Strategies, and Challenges. Int J Biol Macromol. 2025;329:147825. 10.1016/J.IJBIOMAC.2025.147825. 40987370

[30] Hao Z, Kong L, Ruan L, Deng Z. Recent Advances in DNA Origami-Enabled Optical Biosensors for Multi-Scenario Application. *Nanomaterials 2024, Vol. 14, Page* 1968 2024, *14*, 1968. 10.3390/NANO14231968 PMC11643833 39683355

[31] Niu R, Tao M, Chao J. The Application of DNA Origami in Biosensing. Chem 2025. 2025;7:165. 10.3390/CHEMISTRY7050165.

[32] Salim IN, Reay R, Denby C, Halloran C, Ngo TA, Ashley JDNA. Origami and Their Application in Biosensors. Biosens 2026. 2026;16:247. 10.3390/BIOS16050247. PMC13204131 42187443

[33] Yuan C, Zhou F, Xu Z, Wu D, Hou P, Yang D, Pan L, Wang P. Functionalized DNA Origami-Enabled Detection of Biomarkers. ChemBioChem. 2024;25:e202400227. 10.1002/cbic.202400227. 38700476

[34] Li G, Chen C, Li Y, Wang B, Wen J, Guo M, Chen M, Zhang X-B, Ke G. DNA-Origami-Based Precise Molecule Assembly and Their Biological Applications. Nano Lett. 2024;24:11335–48. 10.1021/acs.nanolett.4c03297. 39213537

[35] Wu Y, Wu X, Tian R, Wang Y, Ding B, Jiang Q. Precise Construction of DNA Origami-based Materials for Functional Regulation on Biological Interface. Smart Molecules. 2024;2:e20230032. 10.1002/smo.20230032. PMC12118239 40625526

[36] Purohit B, Vernekar PR, Shetti NP, Chandra P. Biosensor Nanoengineering: Design, Operation, and Implementation for Biomolecular Analysis. Sens Int. 2020;1:100040. 10.1016/j.sintl.2020.100040.

[37] Szabó Á, Szöllősi J, Nagy P. Principles of Resonance Energy Transfer. Curr Protoc. 2022;2:e625. 10.1002/cpz1.625. 36507547

[38] Ha T, Enderle T, Ogletree DF, Chemla DS, Selvin PR, Weiss S. Probing the Interaction between Two Single Molecules: Fluorescence Resonance Energy Transfer between a Single Donor and a Single Acceptor. Proc Natl Acad Sci U S A. 1996;93:6264–8. 10.1073/PNAS.93.13.6264. PMC39010 8692803

[39] Aoki K, Komatsu N, Hirata E, Kamioka Y, Matsuda M. Stable Expression of < scp>FRET Biosensors: A New Light in Cancer Research. Cancer Sci. 2012;103:614–9. 10.1111/j.1349-7006.2011.02196.x. PMC7659274 22188216

[40] Liang P, Li J, Pi Z, Zhang X, Yu X, Lai G. Hyperbranched RCA-Mediated Construction of a Dendritic DNA Nanostructure for Ratiometric Fluorescence Biosensing. Sens Actuators B Chem. 2024;412:135797. 10.1016/j.snb.2024.135797.

[41] Rye HS, Yue S, Wemmer DE, Quesada MA, Haugland RP, Mathies RA, Glazer AN. Stable Fluorescent Complexes of Double-Stranded DNA with Bis-Intercalating Asymmetric Cyanine Dyes: Properties and Applications. Nucleic Acids Res. 1992;20:2803–12. 10.1093/nar/20.11.2803. PMC336925 1614866

[42] Chen C, Midelet C, Bhuckory S, Hildebrandt N, Werts MHV. Nanosurface Energy Transfer from Long-Lifetime Terbium Donors to Gold Nanoparticles. J Phys Chem C. 2018;122:17566–74. 10.1021/acs.jpcc.8b06539.

[43] Maxwell DJ, Taylor JR, Nie S. Self-Assembled Nanoparticle Probes for Recognition and Detection of Biomolecules. J Am Chem Soc. 2002;124:9606–12. 10.1021/ja025814p. 12167056

[44] Selnihhin D, Sparvath SM, Preus S, Birkedal V, Andersen ES. Multifluorophore DNA Origami Beacon as a Biosensing Platform. ACS Nano. 2018;12:5699–708. 10.1021/acsnano.8b01510. 29763544

[45] Lu C, Yang H, Zhu C, Chen X, Chen G. A Graphene Platform for Sensing Biomolecules. Angew Chem Int Ed. 2009;48:4785–7. 10.1002/anie.200901479. 19475600

[46] Jang H, Kim Y-K, Kwon H-M, Yeo W-S, Kim D-E, Min D-H. A Graphene-Based Platform for the Assay of Duplex-DNA Unwinding by Helicase†. Angew Chem. 2010;122:5839–43. 10.1002/ANGE.201001332. PMC7159641 32313315

[47] Trofymchuk K, Kołątaj K, Glembockyte V, Zhu F, Acuna GP, Liedl T, Tinnefeld P. Gold Nanorod DNA Origami Antennas for 3 Orders of Magnitude Fluorescence Enhancement in NIR. ACS Nano. 2023;17:1327–34. 10.1021/acsnano.2c09577. 36594816

[48] Acuna GP, Möller FM, Holzmeister P, Beater S, Lalkens B, Tinnefeld P. Fluorescence Enhancement at Docking Sites of DNA-Directed Self-Assembled Nanoantennas. Sci (1979). 2012;338:506–10. 10.1126/science.1228638. 23112329

[49] Bauch M, Toma K, Toma M, Zhang Q, Dostalek J. Plasmon-Enhanced Fluorescence Biosensors: A Review. *Plasmonics 2013 9:4* 2013, *9*, 781–799. 10.1007/S11468-013-9660-5 PMC4846700 27330521

[50] Choi J-H, Lim J, Shin M, Paek S-H, Choi J-W. CRISPR-Cas12a-Based Nucleic Acid Amplification-Free DNA Biosensor via Au Nanoparticle-Assisted Metal-Enhanced Fluorescence and Colorimetric Analysis. Nano Lett. 2021;21:693–9. 10.1021/acs.nanolett.0c04303. 33346665

[51] Dass M, Gür FN, Kołątaj K, Urban MJ, Liedl TDNA. Origami-Enabled Plasmonic Sensing. J Phys Chem C. 2021;125:5969–81. 10.1021/acs.jpcc.0c11238. PMC8016175 33828635

[52] Close C, Trofymchuk K, Grabenhorst L, Lalkens B, Glembockyte V, Tinnefeld P. Maximizing the Accessibility in DNA Origami Nanoantenna Plasmonic Hotspots. Adv Mater Interfaces. 2022;9:2200255. 10.1002/admi.202200255.

[53] Latorre A, Somoza Á. DNA-Mediated Silver Nanoclusters: Synthesis, Properties and Applications. ChemBioChem. 2012;13:951–8. 10.1002/cbic.201200053. 22508551

[54] Yeh H-C, Sharma J, Han JJ, Martinez JS, Werner JH. A DNA–Silver Nanocluster Probe That Fluoresces upon Hybridization. Nano Lett. 2010;10:3106–10. 10.1021/nl101773c. 20698624

[55] Zhou Z, Du Y, Dong S, Double-Strand DNA-T. Formation of Copper Nanoparticles as Fluorescent Probe for Label-Free Aptamer Sensor. Anal Chem. 2011;83:5122–7. 10.1021/ac200120g. 21612269

[56] Qing Z, He X, Qing T, Wang K, Shi H, He D, Zou Z, Yan L, Xu F, Ye X, et al. Poly(Thymine)-Templated Fluorescent Copper Nanoparticles for Ultrasensitive Label-Free Nuclease Assay and Its Inhibitors Screening. Anal Chem. 2013;85:12138–43. 10.1021/ac403354c. 24236868

[57] Pothoulakis G, Ceroni F, Reeve B, Ellis T. The Spinach RNA Aptamer as a Characterization Tool for Synthetic Biology. ACS Synth Biol. 2014;3:182–7. 10.1021/sb400089c. 23991760

[58] Nilaratanakul V, Hauer DA, Griffin DE. Development of Encoded Broccoli RNA Aptamers for Live Cell Imaging of Alphavirus Genomic and Subgenomic RNAs. Sci Rep. 2020;10:5233. 10.1038/s41598-020-61573-3. PMC7090087 32251299

[59] Diao W, Tang M, Ding S, Li X, Cheng W, Mo F, Yan X, Ma H, Yan Y. Highly Sensitive Surface Plasmon Resonance Biosensor for the Detection of HIV-Related DNA Based on Dynamic and Structural DNA Nanodevices. Biosens Bioelectron. 2018;100:228–34. 10.1016/j.bios.2017.08.042. 28918231

[60] Yang S, Zhao Z, Wang B, Feng L, Luo J, Deng R, Sheng J, Gao X, Xie S, Chen M, et al. Modular Engineering of a DNA Tetrahedron-Based Nanomachine for Ultrasensitive Detection of Intracellular Bioactive Small Molecules. ACS Appl Mater Interfaces. 2023;15:23662–70. 10.1021/acsami.3c02614. 37140536

[61] Wei L, Wang Z, Dong Y, Yu D, Chen Y. Enhanced CRISPR/Cas12a Fluorimetry via a DNAzyme-Embedded Framework Nucleic Acid Substrate. Anal Chem. 2024;96:16453–61. 10.1021/acs.analchem.4c04710. 39367822

[62] Cui J, Han H, Piao J, Shi H, Zhou D, Gong X, Chang J. Construction of a Novel Biosensor Based on the Self-Assembly of Dual-Enzyme Cascade Amplification-Induced Copper Nanoparticles for Ultrasensitive Detection of MicroRNA153. ACS Appl Mater Interfaces. 2020;12:34130–6. 10.1021/acsami.0c06032. 32627523

[63] Zhang S, Huang J, Lu J, Liu M, Li Y, Fang L, Huang H, Huang J, Mo F, Zheng J. A Novel Fluorescent Biosensor Based on Dendritic DNA Nanostructure in Combination with Ligase Reaction for Ultrasensitive Detection of DNA Methylation. J Nanobiotechnol. 2019;17:121. 10.1186/s12951-019-0552-5. PMC6898925 31812164

[64] Zhou Z, Sohn YS, Nechushtai R, Willner IDNA. Tetrahedra Modules as Versatile Optical Sensing Platforms for Multiplexed Analysis of MiRNAs, Endonucleases, and Aptamer–Ligand Complexes. ACS Nano. 2020;14:9021–31. 10.1021/acsnano.0c04031. PMC7467810 32539340

[65] Ahmadi-Sangachin E, Mohammadnejad J, Hosseini M. Fluorescence Self-Assembled DNA Hydrogel for the Determination of Prostate Specific Antigen by Aggregation Induced Emission. Spectrochim Acta Mol Biomol Spectrosc. 2023;303:123234. 10.1016/j.saa.2023.123234. 37582316

[66] Li Y, Wu Y, Xu R, Guo J, Quan F, Zhang Y, Huang D, Pei Y, Gao H, Liu W, et al. In Vivo Imaging of Mitochondrial DNA Mutations Using an Integrated Nano Cas12a Sensor. Nat Commun. 2023;14:7722. 10.1038/s41467-023-43552-0. PMC10673915 38001092

[67] Xue D, Su J, Chen S, Lin F, Wu R, Hong C, Lin L, Chen X. Ultrasensitive Fluorescence Detection of Lung Cancer-Related MiRNAs Based on DNA Tetrahedron and DNAzyme Catalytic Reaction. Anal Chem. 2026;98:2297–304. 10.1021/acs.analchem.5c06315. 41518266

[68] Zhang L, Guo S, Zhu J, Zhou Z, Li T, Li J, Dong S, Wang E. Engineering DNA Three-Way Junction with Multifunctional Moieties: Sensing Platform for Bioanalysis. Anal Chem. 2015;87:11295–300. 10.1021/acs.analchem.5b02468. 26509479

[69] Khajavian Z, Esmaelpourfarkhani M, Ramezani M, Alibolandi M, Abnous K, Taghdisi SMA, Highly Sensitive. Simple and Label-Free Fluorescent Aptasensor for Tobramycin Sensing Based on PicoGreen Intercalation into DNA Duplex Regions of Three-Way Junction Origami. Microchem J. 2021;160:105657. 10.1016/j.microc.2020.105657.

[70] He J-Y, Deng H-L, Shang X, Yang C-L, Zuo S-Y, Yuan R, Liu H-Y, Xu W-J. Modulating the Fluorescence of Silver Nanoclusters Wrapped in DNA Hairpin Loops via Confined Strand Displacement and Transient Concatenate Ligation for Amplifiable Biosensing. Anal Chem. 2022;94:8041–9. 10.1021/acs.analchem.2c01354. 35617342

[71] Yang C, Deng H, He J, Zhang X, Gao J, Shang X, Zuo S, Yuan R, Xu W. Amplifiable Ratiometric Fluorescence Biosensing of Nanosilver Multiclusters Populated in Three-Way-Junction DNA Branches. Biosens Bioelectron. 2022;199:113871. 10.1016/J.BIOS.2021.113871. 34915217

[72] Torelli E, Shirt-Ediss B, Navarro SA, Manzano M, Vizzini P, Krasnogor N. Light-Up Split Broccoli Aptamer as a Versatile Tool for RNA Assembly Monitoring in Cell-Free TX-TL Systems, Hybrid RNA/DNA Origami Tagging and DNA Biosensing. Int J Mol Sci. 2023;24:8483. 10.3390/ijms24108483. PMC10218337 37239830

[73] Xu C, Wang X, Li H, Han C, Wang J, Wang Y, Liu S, Huang J. Branched RCA Coupled with a NESA-Based Fluorescence Assay for Ultrasensitive Detection of MiRNA. New J Chem. 2017;41:5355–61. 10.1039/C7NJ00404D.

[74] Xie Y, Xie G, Yuan J, Zhang J, Yang Y, Yao Y, Wu Y, Bai D, Chen K, Li B, et al. A Novel Fluorescence Biosensor Based on Double-Stranded DNA Branch Migration-Induced HCR and DNAzyme Feedback Circuit for Sensitive Detection of Pseudomonas Aeruginosa (Clean Version). Anal Chim Acta. 2022;1232:340449. 10.1016/j.aca.2022.340449. 36257735

[75] Pan D, Mijit M, Wang H, Sun C, Pingcuo B, Yu Z, Xiong B, Tang X. Rapid Genotyping of FecB Mutation in Sheep Using CRISPR-Cas12a Integrated with DNA Nanotree Biosensing Platform. Biomolecules. 2025;15:315. 10.3390/biom15030315. PMC11940080 40149851

[76] Wu S, Gong M, You Y, Zhu J, Lai G. Amplified Assembly of Silver Nanoclusters-Decorated Hyperbranched DNA Nanostructures at Metal-AIEgen Frameworks for Ratiometric Fluorescence Biosensing of Estrogen Pollutants. Sens Actuators B Chem. 2026;465:140195. 10.1016/j.snb.2026.140195.

[77] Goodman RP, Berry RM, Turberfield AJ. The Single-Step Synthesis of a DNA Tetrahedron. Chem Commun. 2004;40:1372. 10.1039/b402293a. 15179470

[78] Chen Z, Meng C, Wang X, Chen J, Deng J, Fan T, Wang L, Lin H, Huang H, Li S, et al. Ultrasensitive DNA Origami Plasmon Sensor for Accurate Detection in Circulating Tumor DNAs. Laser Photon Rev. 2024;18:2400035. 10.1002/lpor.202400035.

[79] Zhao Z, Xie Z, Chen S, Chen M, Wang X, Yi GA. Novel Biosensor Based on Tetrahedral DNA Nanostructure and Terminal Deoxynucleotidyl Transferase-Assisted Amplification Strategy for Fluorescence Analysis of Uracil-DNA Glycosylase Activity. Anal Chim Acta. 2023;1271:341432. 10.1016/j.aca.2023.341432. 37328254

[80] Zhang K, Huang W, Huang Y, Li H, Wang K, Zhu X, Xie MDNA. Tetrahedron Based Biosensor for Argonaute2 Assay in Single Cells and Human Immunodeficiency Virus Type-1 Related Ribonuclease H Detection in Vitro. Anal Chem. 2019;91:7086–96. 10.1021/acs.analchem.9b00011. 31050888

[81] Xu D, Guo L, Xie Z, He P, Song W, Zhou HA, Label-Free CRISPR. /Cas12a Fluorescent Aptasensor for AFB1 Detection Based on Guanine Nanowire Assisted Three Dimensional DNA Networks with Catalytic Hairpin Assembly. Sens Actuators B Chem. 2025;441:138041. 10.1016/j.snb.2025.138041.

[82] Feng C, Che X, Mao C, Wang B, Zhou Z, Chen G, Zhang P. Enhanced DNAzyme-Based Tetrahedron Amplifier for Ultrasensitive Determination and Intracellular Imaging of Breast Cancer-Related MicroRNAs. ACS Sens. 2025;10:8895–905. 10.1021/acssensors.5c02856. 41217073

[83] Yang Z, Yang B, Wang J, Yu Q, Du W, Meng Y, Li Q, Xu H, Wang W, Lyu JDNA. Tetrahedron-Assisted Catalytic Hairpin Assembly Enables Robust in Situ MiRNA Detection and Imaging in Living Cells and Clinical Samples. Sens Actuators B Chem. 2025;444:138453. 10.1016/j.snb.2025.138453.

[84] Yang Y, Yang J, Yang Y, Ji X, Li W, Wang F, Xie C, He Z. CRISPR/Cas13a Catalyzed Self-Assembly of Quantum Dot-DNA Hydrogel for MicroRNA Assay. Sens Actuators B Chem. 2023;393:134249. 10.1016/j.snb.2023.134249.

[85] Wang X, Wang Z, Dong F, Yang D, Yin L, Han L. Exploration of Water-Soluble Natural AIEgens Boosting Label-Free Turn-on Fluorescent Sensing in a DNA Hydrogel. Anal Chem. 2023;95:13864–71. 10.1021/acs.analchem.3c02004. 37643162

[86] Liu C, Zhu Q, Kuang J, Zhou X, Ma Z, Huang C, Song J, Shen W, Lee HK, Tang SDNA. Hydrogel-Based Fluorescence Biosensor Packed in a Three-Dimensional-Printed Microextractor for Direct Tracking of Free Antibiotic Resistance Genes in Wastewater Treatment Plants. ACS Sens. 2026;11:4024–32. 10.1021/acssensors.6c00391. 42012074

[87] Wang X, Zeng Y, Qi P, Wang P, Wang W, Wang D. Self Assembling Fluorescent Biosensing System Based on Rolling Circle Amplification Combined with DNA Templated Quantum Dots for Detecting Microbial DNA. Sens Actuators B Chem. 2026;450:139286. 10.1016/j.snb.2025.139286.

[88] Zhang Z, Ma J, Zhang G, Ding X, Zhang R, Zhou T, Wang X, Wang F. Large-Scale DNA Nanoarrays with a Controllable Fluorescence Switch Constructed by RCA Simplified Origami. Langmuir. 2020;36:10989–95. 10.1021/acs.langmuir.0c01821. 32838532

[89] Li Y, Wu Y, Zheng Z, Wu Y, Zhang Y, Zhang J, Quan F, Zhao W, Xu R, Li Y, et al. Renal Clearable CRISPR Nanosensor Targeting Mitochondrial DNA Mutation for Noninvasive Monitoring of Tumor Progression and Metastasis. Sci Adv. 2025;11:eadz4594. 10.1126/sciadv.adz4594. PMC12637282 41270157

[90] Zhang Z, Wang J, Li C, Sun H, Bu S, Jia Q, Wan Y, Zhao Y, Zhou H, Hao Z, et al. An Ultrasensitive Biosensor for H1N1 Virus Coupled with 3D Spherical DNA Nanostructure and CRISPR-Cas12a. Spectrochim Acta Mol Biomol Spectrosc. 2026;346:126905. 10.1016/j.saa.2025.126905. 40921108

[91] Sui Z, Chen B, Zhao J, Yang H, Guo L, Xu J. Dual-Accelerated Signal Amplification in Biosensing via Spatial Confining Catalytic Hairpin Assembly-Activated Spherical CRISPR/Cas12a System for Trans-Cleavage of Hairpin DNA Reporters. Anal Chem. 2025;97:4668–77. 10.1021/acs.analchem.4c07111. 39982896

[92] Liang G-X, Ye S-Y, Yu H-M, Zhao K-R, Liu P-F, Liu Z-J, Wang LA. Potent Fluorescent Biosensor Integrating 3D DNA Walker with Localized Catalytic Hairpin Assembly for Highly Sensitive and Enzyme-Free Zika Virus Detection. Sens Actuators B Chem. 2022;354:131199. 10.1016/j.snb.2021.131199.

[93] Xiong E, Zhen D, Jiang L, Zhou X. Binding-Induced 3D-Bipedal DNA Walker for Cascade Signal Amplification Detection of Thrombin Combined with Catalytic Hairpin Assembly Strategy. Anal Chem. 2019;91:15317–24. 10.1021/acs.analchem.9b04987. 31710462

[94] Yang L, Fang J, Li J, Ou X, Zhang L, Wang Y, Weng Z, Xie G. An Integrated Fluorescence Biosensor for MicroRNA Detection Based on Exponential Amplification Reaction-Triggered Three-Dimensional Bipedal DNA Walkers. Anal Chim Acta. 2021;1143:157–65. 10.1016/j.aca.2020.11.043. 33384113

[95] Li D, Yang E, Luo Z, Xie Q, Duan Y. An Enzyme-Mediated Universal Fluorescent Biosensor Template for Pathogen Detection Based on a Three-Dimensional DNA Walker and Catalyzed Hairpin Assembly. Nanoscale. 2021;13:2492–501. 10.1039/D0NR07593K. 33471006

[96] Shahbazi E, Mollasalehi H, Minai-Tehrani DA, Gold Nanoparticle. Conjugated Single-Legged DNA Walker Driven by Catalytic Hairpin Assembly Biosensor to Detect a Prokaryotic Pathogen. Sci Rep. 2024;14:22980. 10.1038/s41598-024-74227-5. PMC11449931 39363058

[97] Wang K, Yan X, Wu J, Qi J, Ning M, Li M, Sun R, Wang Z, Yuan Y, Yue T. A Fluorescent Aptasensor for Deoxynivalenol Detection Based on Nb.BbvCI-Assisted Targeted-Responsive Three-Way Junctions Integrated DNA Walking Machine. Food Chem. 2025;467:142365. 10.1016/j.foodchem.2024.142365. 39657483

[98] Yu M, Yang Z, Tan M, Chen J, Shen W, Wang J, Zhao X, Chen G, Zhang Z, Liu Y. Catalytic Hairpin Assembly RNA-Cleaving DNAzyme Junction Mediated Circular-CrRNA Regulated DNA Walker for Sensitive and Reliable Atherosclerosis Related MicroRNA Analysis. Microchem J. 2026;226:118409. 10.1016/j.microc.2026.118409.

[99] Yin L-K, Huang R, Wang Z-L, Yang C, Mi X, Zhan H-Y, Zhang Z-Q. A DNA Walker-Based Biosensor for Ultrasensitive HIV DNA Detection. Sens Actuators Rep. 2026;11:100435. 10.1016/j.snr.2026.100435.

[100] Hong F, Zhang F, Liu Y, Yan HDNA, Origami. Scaffolds for Creating Higher Order Structures. Chem Rev. 2017;117:12584–640. 10.1021/acs.chemrev.6b00825. 28605177

[101] Domljanovic I, Kocabey S, Acuna P, Ruegg G. Enhancing the MiRNA Detection Sensitivity of DNA Origami Book Biosensors Using Lock Modifications and a Polymer Additive. Small. 2025;21:e12100. 10.1002/smll.202512100. PMC12747634 41235702

[102] Pfeiffer M, Trofymchuk K, Ranallo S, Ricci F, Steiner F, Cole F, Glembockyte V, Tinnefeld P. Single Antibody Detection in a DNA Origami Nanoantenna. iScience. 2021;24:103072. 10.1016/j.isci.2021.103072. PMC8449233 34568793

[103] Choi Y, Schmidt C, Tinnefeld P, Bald I, Rödiger SA. New Reporter Design Based on DNA Origami Nanostructures for Quantification of Short Oligonucleotides Using Microbeads. Sci Rep. 2019;9:4769. 10.1038/s41598-019-41136-x. PMC6423227 30886341

[104] Trofymchuk K, Glembockyte V, Grabenhorst L, Steiner F, Vietz C, Close C, Pfeiffer M, Richter L, Schütte ML, Selbach F et al. Addressable Nanoantennas with Cleared Hotspots for Single-Molecule Detection on a Portable Smartphone Microscope. *Nature Communications 2021 12:1* 2021, *12*, 950-, 10.1038/s41467-021-21238-9 PMC7878865 33574261

[105] Yaadav R, Trofymchuk K, Dass M, Behrendt V, Hauer B, Schütz J, Close C, Scheckenbach M, Ferrari G, Mäurer L, et al. Bringing Attomolar Detection to the Point-of-Care with Nanopatterned DNA Origami Nanoantennas. Adv Mater. 2025;37. 10.1002/adma.202507407. PMC12510279 40714742

[106] Kaur V, Kaur C, Sen T. Single-Molecule Fluorescence Enhancement Based Detection of ATP Using DNA Origami-Assembled Au@Ag Nanostar Optical Antennas. J Phys Chem C. 2023;127:7308–18. 10.1021/acs.jpcc.3c00020.

[107] Corduri N, Veedu MK, Yu Y, Zou Y, Liu J, Garoli D, Acuna GP, Wenger J, Kołątaj K. DNA-Origami‐Assembled Rhodium Nanoantennas for Deep‐UV Label‐Free Single‐Protein Detection. Adv Funct Mater. 2026;36:e32006. 10.1002/adfm.202532006.

[108] Jepsen MDE, Sørensen RS, Maffeo C, Aksimentiev A, Kjems J, Birkedal V. Single Molecule Analysis of Structural Fluctuations in DNA Nanostructures. Nanoscale. 2019;11:18475–82. 10.1039/C9NR03826D. PMC6825326 31577314

[109] Domljanovic I, Loretan M, Kempter S, Acuna GP, Kocabey S, Ruegg CDNA. Origami Book Biosensor for Multiplex Detection of Cancer-Associated Nucleic Acids. Nanoscale. 2022;14:15432–41. 10.1039/D2NR03985K. PMC9612396 36219167

[110] Walter H-K, Bauer J, Steinmeyer J, Kuzuya A, Niemeyer CM, Wagenknecht H-A. DNA Origami Traffic Lights with a Split Aptamer Sensor for a Bicolor Fluorescence Readout. Nano Lett. 2017;17:2467–72. 10.1021/acs.nanolett.7b00159. 28249387

[111] Tang MSL, Shiu SC-C, Godonoga M, Cheung Y-W, Liang S, Dirkzwager RM, Kinghorn AB, Fraser LA, Heddle JG, Tanner JA. An Aptamer-Enabled DNA Nanobox for Protein Sensing. Nanomedicine. 2018;14:1161–8. 10.1016/j.nano.2018.01.018. 29410111

[112] Tsang E, Lund LM, Birkedal V, Gothelf KV. Single-Molecule Nucleic Acid Detection with a Reconfigurable Rotating DNA Origami Nanodevice. ACS Nano. 2026;20:4607–16. 10.1021/acsnano.5c22080. 41586571

[113] Ochmann SE, Joshi H, Büber E, Franquelim HG, Stegemann P, Saccà B, Keyser UF, Aksimentiev A, Tinnefeld PDNA. Origami Voltage Sensors for Transmembrane Potentials with Single-Molecule Sensitivity. Nano Lett. 2021;21:8634–41. 10.1021/acs.nanolett.1c02584. 34662130

[114] Ochmann SE, Schröder T, Schulz CM, Tinnefeld P. Quantitative Single-Molecule Measurements of Membrane Charges with DNA Origami Sensors. Anal Chem. 2022;94:2633–40. 10.1021/acs.analchem.1c05092. 35089694

[115] Büber E, Yaadav R, Schröder T, Franquelim HG, Tinnefeld PDNA. Origami Vesicle Sensors with Triggered Single-Molecule Cargo Transfer. Angew Chem Int Ed. 2024;63:e202408295. 10.1002/anie.202408295. PMC11586697 39248369

[116] Büber E, Schröder T, Scheckenbach M, Dass M, Franquelim HG, Tinnefeld PDNA. Origami Curvature Sensors for Nanoparticle and Vesicle Size Determination with Single-Molecule FRET Readout. ACS Nano. 2023;17:3088–97. 10.1021/acsnano.2c11981. 36735241

[117] Shahhosseini M, Beshay PE, Akbari E, Roki N, Lucas CR, Avendano A, Song JW, Castro CE. Multiplexed Detection of Molecular Interactions with DNA Origami Engineered Cells in 3D Collagen Matrices. ACS Appl Mater Interfaces. 2022;14:55307–19. 10.1021/acsami.2c07971. PMC9785045 36509424

[118] Dai J, Xing C, Lin Y, Huang Y, Yang Y, Chen Z, Lu C, Yang H. Localized DNA Catalytic Hairpin Assembly Reaction on DNA Origami for Tumor-Associated MicroRNA Detection and Imaging in Live Cells. Sens Actuators B Chem. 2021;344:130195. 10.1016/j.snb.2021.130195.

[119] Cervantes-Salguero K, Freeley M, Chávez JL, Palma M, Single-Molecule DNA. Origami Aptasensors for Real-Time Biomarker Detection. J Mater Chem B. 2020;8:6352–6. 10.1039/D0TB01291B. 32716449

[120] Zhou L, Xiong Y, Dwivedy A, Zheng M, Cooper L, Shepherd S, Song T, Hong W, Le LTP, Chen X et al. Bioinspired Designer DNA NanoGripper for Virus Sensing and Potential Inhibition. *Sci. Robot.* 2024, *9*, 10.1126/scirobotics.adi2084. PMC11750070 39602515

[121] Rodríguez-Franco HJ, Hendrickx PBM, Bastings MMC. Tailoring Origami Protection: A Study of Oligolysine-PEG for Improved Colloidal, Structural, and Functional Integrity. ACS Polym Au. 2025;5:35–44. 10.1021/ACSPOLYMERSAU.4C00085. PMC11826485 39958528

[122] Moghadam MG, Douglas TR, Alexander S, Fiddes LK, Tilstra G, Khan OF, Chou LY. .T. PEG-Grafted Oligolysines DNA Origami While Receptor-Specific Cell Binding. J Am Chem Soc. 2025;147:26748–61. 10.1021/JACS.5C07566. 40689691

[123] Goodrum R, Li H. Advances in Three Dimensional Metal Enhanced Fluorescence Based Biosensors Using Metal Nanomaterial and Nano-Patterned Surfaces. Biotechnol J. 2024;19:2300519. 10.1002. /BIOT.202300519;CTYPE:STRING:JOURNAL. 10.1002/biot.202300519 37997672

[124] Zaza C, Chiarelli G, Zweifel LP, Pilo-Pais M, Sisamakis E, Barachati F, Stefani FD, Acuna GP. Super‐Resolved FRET Imaging by Confocal Fluorescence‐Lifetime Single‐Molecule Localization Microscopy. Small Methods. 2023;7:2201565. 10.1002/smtd.202201565. 37132097

[125] Linko V, Keller A. Stability of DNA Origami Nanostructures in Physiological Media: The Role of Molecular Interactions. Small. 2023;19:2301935. 10. 1002/SMLL.202301935;CTYPE:STRING:JOURNAL. 10.1002/smll.202301935 37093216

[126] Keller A, Linko V. Challenges and Perspectives of DNA Nanostructures in Biomedicine. Angew Chem Int Ed. 2020;59:15818–33. 10. 1002/ANIE.201916390;ISSUE:ISSUE:DOI. 10.1002/anie.201916390 PMC7540699 32112664

[127] Matulakul P, Phanchai W, Thonghlueng J, Kasemsuk N, Panitanarak U, Srisodaphol W, Wiratchawa K, Wanna Y, Intharah T, Namdee K, et al. Machine Learning-Assisted Detection of Single-Point Mutations via DNA-Templated Gold Nanoparticle Growth. Nanoscale. 2025;17:21042–55. 10.1039/D5NR01187F. 40859743

[128] Kim MJ, Yum K, Kim D, Jang I, Park A, Jung MJ, Lee GD, Jin JO, Im W, Lee JB et al. Quantum Dot–DNA Microsphere Aptamer Biosensor with AI-Assisted Structural Modeling for Rapid Detection of the Lung Cancer Biomarker USE1. *Journal of Nanobiotechnology 2026 24:1* 2026, *24*, 470-, 10.1186/S12951-026-04384-4 PMC13200455 41981644

[129] Ijäs H, Trommler J, Nguyen L, van Rest S, Nickels PC, Liedl T, Urban MJ. DNA Origami Signal Amplification in Lateral Flow Immunoassays. *Nature Communications 2025 16:1* 2025, *16*, 3216-, 10.1038/s41467-025-57385-6 PMC11971289 40185718

